# High-resolution phylogenetic and population genetic analysis of microbial communities with RoC-ITS

**DOI:** 10.1038/s43705-022-00183-8

**Published:** 2022-10-10

**Authors:** Douglas B. Rusch, Jie Huang, Chris Hemmerich, Matthew W. Hahn

**Affiliations:** 1 Center for Genomics and Bioinformatics, Indiana University, Bloomington, IN 47405 USA; 2 Department of Biology, Indiana University, Bloomington, IN 47405 USA; 3 Department of Computer Science, Indiana University, Bloomington, IN 47405 USA

**Keywords:** Biodiversity, Microbial ecology

## Abstract

Microbial communities are inter-connected systems of incredible complexity and dynamism that play crucial roles in health, energy, and the environment. To better understand microbial communities and how they respond to change, it is important to know which microbes are present and their relative abundances at the greatest taxonomic resolution possible. Here, we describe a novel protocol (RoC-ITS) that uses the single-molecule Nanopore sequencing platform to assay the composition of microbial communities at the subspecies designation. Using rolling-circle amplification, this methodology produces long-read sequences from a circular construct containing the complete 16S ribosomal gene and the neighboring internally transcribed spacer (ITS). These long reads can be used to generate a high-fidelity circular consensus sequence. Generally, the ribosomal 16S gene provides phylogenetic information down to the species-level, while the much less conserved ITS region contains strain-level information. When linked together, this combination of markers allows for the identification of individual ribosomal units within a specific organism and the assessment of their relative stoichiometry, as well as the ability to monitor subtle shifts in microbial community composition with a single generic assay. We applied RoC-ITS to an artificial microbial community that was also sequenced using the Illumina platform, to assess its accuracy in quantifying the relative abundance and identity of each species.

## Introduction

The bacterial ribosomal RNA operon (rrn) typically produces a polycistronic precursor RNA containing the 5S, 16S, and 23S rRNA genes along with an internal transcribed spacer (ITS) region [1]. While there are exceptional circumstances, for example with the recent observation of 16S genes unlinked from the other ribosomal genes [2], these cases are the exception rather than the rule. After expression, the precursor RNA is cleaved by a cascade of RNases to generate the individual gene products, and the ITS region is degraded [3]. The individual genes produce key RNA products that are involved in protein production and thus these genes, or their homologs, are found in all free-living organisms. Individual genomes may have one or more ribosomal operons [4] and the presence of multiple rrn copies is thought to allow cells to quickly respond to favorable growth conditions by increasing growth rates [5]. Although multiple rrn copies are often homogenized by concerted evolution, there are many instances of intragenomic rRNA heterogeneity [4, 6].

The 16S gene has become the focus of modern microbial phylogenetics by virtue of its length and mix of highly conserved and variable regions. The conserved regions make it amenable for polymerase chain reaction (PCR) amplification, while the variable regions make it a useful phylogenetic marker [7]. As a result, it has a long history of use in phylogenetics and is widely used to taxonomically survey microbial populations [8, 9]. While longer, the 23S gene has a lower density of informative markers and is therefore rarely used for general phylogenetic purposes in eubacteria; [7] the 5S gene is small and relatively rapidly evolving, so has seen occasional use as a phylogenetic marker [10–12]. The ITS region is the most rapidly evolving part of the rrn [13], likely because it has no defined functional role except for the occasional tRNA genes found within) [1]. This poor conservation and variability in length has made it difficult to use as a phylogenetic marker. However, the position of the ITS region between the highly conserved portions of the 16S and 23S genes means that it can be readily amplified with conserved primers. It has therefore been used as a high-resolution phylogenetic marker [14–17] or more generically as a DNA fingerprinting in a technique called ARISA [18–20].

Due to its ease of amplification and the density of informative sites, the 16S gene has long been the primary target for phylogenetic and taxonomic study. The full-length 16S sequence can be used for phylogenetic resolution down to the species level [21] and can be readily acquired using Sanger sequencing. As 454 [22, 23] and later Illumina [24] short-read sequencing technologies became available at much lower costs per base, 16S sequencing shifted to focus on individual or small subsets of the nine recognized variable regions [22, 25]. Depending on the organisms involved, this smaller number of regions was sufficient to classify microbes taxonomically to the genus or species level but lacked the phylogenetic resolution of the full-length gene. Recently, strategies that look at multiple variable regions in parallel have been developed to improve the resolution of these variable region-based approaches [26]. On the other hand, metagenomic studies of random genomic regions have revealed tremendous genetic and functional diversity within species [27–29], indicating that short-read sequencing of 16S fails to reflect much of the within-species variation. However, the low cost of short-read sequencing platforms resulted in an explosion of microbial data, along with new databases and tools for analyzing, profiling, and comparing these enormous datasets [30, 31].

New developments in sequencing technologies have ushered in attempts to combine quantity with quality and to recover the richness of the full-length 16S gene and beyond, including the entire rRNA and various portions of it including the ITS, 5S and 23S genes [32–39]. Long-read, single molecule sequencing methods including the Pacific Biosciences and Oxford Nanopore Technologies sequencing platforms have made it possible to sequence thousands and even tens of thousands of base pairs in with a single read [40, 41]. These sequencing techniques have a higher error rate than Illumina short reads, but recent innovations both in how the sequencing is performed and in the reliability of the base calling have greatly improved the quality of the reads. PacBio HiFi reads use a circular consensus strategy to get 99% accuracy on inserts from 15 to 20 kb. Creative use of Oxford Nanopore Technologies (hereafter “Nanopore”) long reads to repetitively sequence a circularized template have similarly resulted in “consensus reads” with reduced error rates; [19, 42–46] on-going improvements to Nanopore base-calling software (e.g., guppy) [47] have achieved 99% accuracy on single pass sequencing data.

Here, we describe a new high-throughput sequencing strategy that relies on rolling-circle amplification coupled with Nanopore long-read single molecule sequencing to capture the entire 16S and ITS region. By sequencing the entire 16S region our method provides high-quality phylogenetic information, including resolution at the species level. The inclusion of the ITS region also allows for the ability to distinguish between sub-species including among individual rrn copies within a genome. Together, these tools allow for resolution between microbial strains, leading to more complete characterization of microbial populations and their dynamics. We describe the steps necessary to prepare and sequence a library of reads using our method, which we call RoC-ITS (pronounced like the word rockets), and detail a computational pipeline that can quickly and effectively analyze the data. We applied RoC-ITS to an artificial community of eubacteria that have also been sequenced with Illumina short-reads in order to demonstrate its effectiveness.

## Methods

**Fig. 1:**
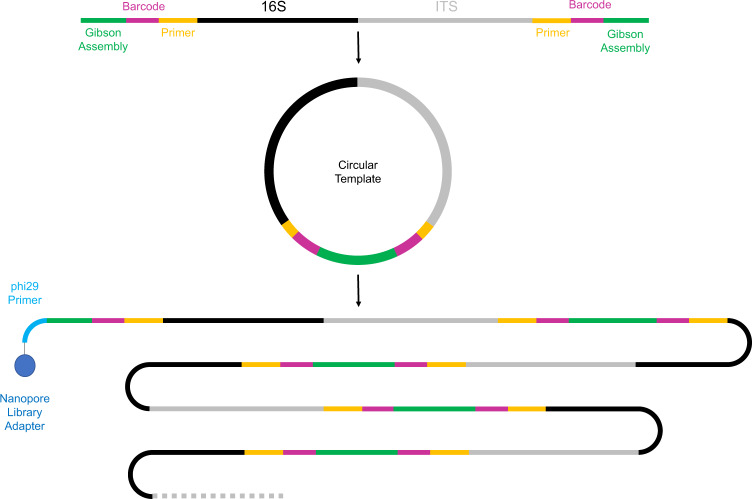
Diagram illustrating RoC-ITS strategy. The 16S-ITS region is isolated using PCR. Gibson Assembly is then used to circularize the PCR products. A long single-stranded DNA product encompassing multiple iterations around the circular template is then generated using the phi29 polymerase. The linear product is finally modified by constructing a double-stranded A-tailed end (Nanopore Library Adapter). At this point, the product is ready for normal library construction using a Nanopore DNA library kit.

The RoC-ITS approach (Fig. 1) involves multiple amplification, circularization, and linearization steps. We describe each of these in turn, followed by additional methods used in this study. Table 1 contains a glossary defining several terms commonly used in the text.

**Table 1:** Definitions of commonly used terminology.

Term	Definition
16S-ITS Region	An instance of the 16S ribosomal RNA and its adjacent ITS region
16S-ITS Product	The PCR product consisting of an instance of the 16S and ITS region defined by the RoC-ITS 27F and RoC-ITS 189R primers
V4 Region	The 4th variable region of the 16S gene as defined by the Earth Microbiome F515/R806 primers
RoC-ITS Consensus Sequence	Consensus sequence derived from multiple independent RoC-ITS sequences
RoC-ITS Sequence	Consensus sequence derived from the amplification and iterated sequencing of a specific 16S-ITS region
RoC-ITS Read	A long Nanopore read derived from a circularized 16S-ITS product consisting of 0 or more sub-reads
Sub-Read	An individual 16S-ITS product found in a RoC-ITS read
Splint DNA	Derived from the lambda virus genome, a sequence used to circularize the 16S-ITS products
ITS	Internally transcribed spacer region; part of the ribosomal RNA operon
ASV	Amplicon sequencing variants; unique sequences identified by DADA2 here derived from the V4 region
Set of Sub-Reads	Multiple sub-reads derived from a single RoC-ITS read
MSA	Multiple sequence alignment
rrn	Ribosomal RNA operon including the 16S and 23S genes flanking the ITS region

### RoC-ITS: PCR amplification

RoC-ITS uses PCR to amplify the 16S-ITS region(s) from an extracted DNA sample. The RoC-ITS 27F 16S primer (5′-AATGATACGGCGACCACCGAGATNNNNNAGAGTTTGATCMTGGCTCAG-3′) was derived from the 27F 16S primer [48, 49] while the RoC-ITS 189R 23S primer (5′-ATGGAAGACGCCAAAAACATAAAGGCTGCNNNNNTACTDAGATGTTTCASTTC-3′) was derived from the 189R primer [50]. In combination, this primer pair should amplify the 16S-ITS regions from eubacterial organisms. Both primers contain a 5-base molecular barcode and have a unique 5′ sequence (underlined; referred to as RoC-ITS 27F Unique and RoC-ITS 189R Unique, respectively). PCR reactions contained 20 ng input DNA in 12.5 μl of KAPA 2X Master Mix (KAPA HiFi HotStart ReadyMix. Catalog number: KK2602. Vendor: Roche Diagnostic), 10 μM forward primer and/or 10 μM reverse primer, and nuclease-free water to bring the final volume to 25 μl.

The overall PCR recipe is given in Supplemental Table 1. Briefly, the first round of PCR added a molecular barcode to the 16S side of the template. The 16S-ITS product was then cleaned with a 0.5x bead cleanup (HighPrepTM PCR paramagnetic bead solution. Vendor: MAGBIO. Catalog number: AC-60050 using a MagStrip Magnet Stand 10. Vendor: MAGBIO. Catalog number: MBMS-10) to remove any unused primer. The second round of PCR and cleanup similarly added the molecular barcode on the 23S end of the molecule. Subsequent rounds of PCR used only the outer (underlined) sequences to amplify only those products that possessed both molecular barcodes. We examined the PCR product on an Agilent TapeStation using a D5000 tape to ensure the bulk of the product was between 2000 and 3500 bp in length.

### RoC-ITS: splint amplification

A splint molecule containing part of the lambda phage genome was generated via PCR similarly to the protocol of Volden et al. [51]. This splint was 386 bp long and has sequence identity with the RoC-ITS 27F Unique and RoC-ITS 189R Unique primers. The splint was generated by PCR with two primers, Lambda_F_23S CTTTATGTTTTTGGCGTCTTCCATAAAGGGATATTTTCGATCGCTTG, where the underlined portion matches the RoC-ITS 189 R Unique primer, and the Lambda_R_16S ATCTCGGTGGTCGCCGTATCATTTGAGGCTGATGAGTTCCATATTTG, that matches the RoC-ITS 27 F Unique primer. The PCR was carried out with 1 μl of Lambda DNA (20 ng/μl; Catalog number: N3011S. Vendor: New England Biolabs Inc), 12.5 μl of KAPA 2X Master Mix, 1 μl of 10 μM Lambda_F_23S and the Lambda_R_16S primers, and nuclease-free water to bring it to its final volume (25 μl). The PCR recipe for this step is shown in Supplementary Table 2. Following PCR, the product was eluted in 30 μL of nuclease-free water after a 1.2× bead cleanup.

### RoC-ITS: circularization

The linear RoC-ITS product was circularized with a DNA splint that matches the RoC-ITS 27F Unique and RoC-ITS 189r Unique primers using Gibson assembly [52]. In a 20 μl reaction, 150 ng of RoC-ITS product and splint DNA were combined with 10 μl of Gibson Assembly 2X Master mix (Catalog number: E2611S Vendor: New England Biolabs Inc) and incubated at 50 °C for 60 min. The reaction was bead-cleaned with 1.0x bead ratio (HighPrepTM PCR paramagnetic bead solution. Vendor: MAGBIO. Catalog number: AC-60050) and eluted in 42 μl of nuclease-free water. Linear molecules in the reaction mixture were then degraded using an exonuclease with the reaction carried out in a 50 μl volume: 2 µl of 25 mM ATP solution, 5 µl of 10X reaction buffer and 1 µl of Plasmid-Safe DNASe at 37 °C for 30 min. The circularized reaction product was isolated with a 1.0× bead cleanup and eluted in 12 μl of nuclease-free water.

### RoC-ITS: rolling circle linearization

The circularized RoC-ITS product was converted into a long single-stranded linear product using the phi29 polymerase (Fig. 1). The phi29 initiation primer (5′-CGCCAGGGTTTTCCCAGTCACGACGAAGACGCCAAAAACATAAAG-3′) has a unique 5′ end (underlined) that does not match the circular template; it was used during the subsequent Nanopore library construction step. The 3′ end of the splint-primer was designed to anneal to the region adjoining the splint DNA adjacent to the RoC-ITS 189r Unique primer and should occur only once per circularized molecule (Fig. 1). This means that only a single product should be produced per circularized molecule, without the usual complications (i.e., branched products) associated with phi29 hexanucleotide-based amplification procedures [51]. The length of the phi29 product is dependent on the stability and processivity of the polymerase and independent of the size of the plasmid. With only a single priming site per molecule, the stoichiometry of the different rrns should be maintained regardless of the length of the ITS region or PCR product. Production of the final linear product was performed in five parallel reactions to generate enough material for subsequent library production. Each reaction was performed in 50 µl at 30 °C overnight with 2 µl of circulated DNA with 2.5 μL of 10 μM (each) dNTPs (Catalog number: N0446S Vendor: New England Biolabs Inc), 2.5 μL Rolling cycle primer (10 μM), 37 μL ultrapure water, 5 μL of 10× Phi29 Buffer, and 1 μL of Phi29 (Catalog number: M0269L Vendor: New England Biolabs Inc). The combined products were bead cleaned at a 0.5× bead ratio and eluted in 10 μl of nuclease-free water.

### RoC-ITS: nanopore library construction and sequencing

Oligos A (5′-**GGCTTCTTCTTGCTCTTAGG**-3′) and B (5′-GTCGTGACTGGGAAAACCCTGGCG**CCTAAGAGCAAGAAGAAGCC**A-3′) are designed to anneal to the phi29 initiation primer (underlined; see Rolling Circle Linearization step) and to each other (bold) to generate an A-overhang required for the Nanopore library kit to function (Fig. 1). This configuration of primers was used to dictate which strand of the library will be preferentially sequenced by the Nanopore flow cell. The annealing buffer (10 mM Tris-HCl pH7.5, 50 mM NaCl) containing 1.4 μM each of oligoA and oligoB is heated to 95 °C for 2 min and allowed to slowly cool to room temperature. The annealed oligoA/B product was then covalently linked to the rolling circle linear product with a ligation reaction: 9.5 µl of cleaned rolling circle product with 1 μL of annealed oligoA/B adaptor, 3.0 μL of NEBNext Quick Ligation Reaction Buffer and 1.5 μL of T4 DNA Ligase (NEBNext Quick Ligation Module; Catalog number: E6056. Vendor: New England Biolabs Inc) incubated at room temperature for 10 min. The oligoA/B adapters were removed from the product with a 1x bead cleanup and eluted into 60 μL of nuclease-free water. After the A and B oligos had been annealed and ligated to the linear single-stranded DNA, a Nanopore DNA library was generated using the Nanopore-provided kit using the following protocol: 60 μL of cleaned adaptor-ligated DNA with 25 μL of ligation buffer (LNB), 10.0 μL of NEBNext Quick T4 DNA ligase and 5 μL of Adapter Mix (AMX) (LNB and AMX are provided in Nanopore SQK-LSK109) and incubated at room temperature for 10 min. The DNA library was purified with a 0.4x bead cleanup and eluted in 15 μL of elution buffer (from Nanopore SQK-LSK109 kit) and was designed to then run Nanopore SQK-LSK109 flow cell R9.4.1 following the prescribed Nanopore protocol. For the data described herein, we reused a previously used but cleaned flow cell of this type (often referred to as a spent flow cell). Spent flow cells usually produce less than 10% of the reads that a new flow cell would produce, however, the data seem to be of comparable quality, so we used this strategy as a cost-effective method for protocol development. Base calling was performed using the Oxford Nanopore guppy_basecaller (version 5.0.7) using a GP100GL video card (Tesla P100 PCIe 16GB, rev a1; NVIDIA Corporation, CA, USA) to generate the RoC-ITS reads. The length of pass quality Nanopore reads is shown in Supplementary Fig. 1. The resulting fastq files were converted to fasta files using vsearch (v2.14.2) [53].

### Processing RoC-ITS reads into RoC-ITS sequences

Individual Nanopore reads have a higher error rate than Illumina reads, with much more frequent insertions and deletions. To identify individual 16S-ITS products, we constructed a DNA HMM using the HMMer software (hmmsearch v3.2.1 with default parameters) to identify the splint DNA (above) and neighboring PCR primer sites along with the molecular barcodes [54]. This HMM was used to identify and remove the splint region thus breaking the long RoC-ITS read into sub-reads; each sub-read should contain a single instance of the 16S-ITS element including the 5 bp random barcodes. Only sub-reads in the expected size range of 1500–3500 bps in length were considered valid and used for further analysis (size distribution of sub-reads is available in Supplementary Fig. 2). The process of extracting the sub-reads was performed with custom python scripts using the output of hmmsearch. Each joint sequence is flanked by a 5-nucleotide random barcode; we required each barcode to be the same for each valid sub-read extracted from the longer nanopore read. For both the 5′ and 3′ barcode, MAFFT was used to build an alignment and biopython was used to generate a gapped consensus with threshold of –barcode-consensus-cutoff. Gapped positions were removed from the consensus and if the resulting sequences were more than five bases, the read is discarded for containing mixed barcodes. Once identified, the barcodes are clipped from the sub-reads and excluded from any subsequent analysis steps.

The sub-reads were further refined with CONSENT-correct [55]. Each set of sub-reads from a single nanopore read was then converted into a multiple sequence alignment using the prank alignment software (http://wasabiapp.org/software/prank/) resulting in a multiple sequence alignment (MSA) file. The MSA was then further refined using probcons (version 1.1) [56] to resolve small issues with consistency in the prank MSA (see the supplemental document for additional details and an example). This improved MSA was then used to generate a consensus sequence, hereafter referred to as a RoC-ITS sequence, that can be analyzed further. Investigating resulting alignments shows that occasionally a read will have one or more sub-reads that differ considerably from the other sub-reads, even after removing conflicting barcodes in an earlier step. To identify these cases, a consensus was built for the alignment using the most common character (base or gap) present in each column. Each sub-read was then scored against this consensus, receiving 1 point for each column where the sub-read matches the consensus, 0.5 points for each column where there is a gap in the sub-read and there was a base called in the consensus, and 0 points for each column that did not a match the options already listed. If the score for a sub-read was less than 65% of the alignment length, that sub-read was discarded. This process is repeated iteratively until either no sub-reads are removed or three or fewer sub-reads remain. Any gaps were removed to produce the final RoC-ITS sequence. To ensure higher quality results, only RoC-ITS sequences derived from five or more final sub-reads were used in subsequent analyses.

The number of sub-reads that contribute to the generation of a RoC-ITS sequence is recorded in the definition line, with each additional sub-read increasing our confidence in the resulting RoC-ITS sequence (lower error rates). Unless otherwise stated, we relied on RoC-ITS sequences derived from five or more sub-reads. This required the length of a good RoC-ITS read to be 10 kb or larger given the typical size of the 16S-ITS region and allowing for the length of the joint sequence. Given the length distribution of the RoC-ITS sequences (Supplementary Fig. S1) this is the single leading cause to sequences being filtered out of our analysis pipeline.

### Construction of the artificial community

At our request, an artificial community was constructed from a set of one to two dozen DNAs from distinct isolates and provided blindly to our team for our method testing purposes. As per our request, the DNA from each isolate was diluted such that the abundance of the different isolates was spread over several orders of magnitude prior to being combined as might be seen in naturally stratified microbial communities. This artificial community was used for the amplification of the V4 region as well as the 16S-ITS region targeted by the RoC-ITS primers.

### Processing artificial community RoC-ITS sequences

RoC-ITS sequences derived from the artificial microbial community were analyzed with a series of filtering and clustering steps before generating phylogenetic trees to examine the rrn structure by genera. A set of high confidence RoC-ITS sequences (*N* = 583) derived from 15 or more sub-reads were used to extract the 16 S gene defined as the bases between the 27F and 1492R [57] inclusive based on strict pattern matching to the universal primers (*N* = 235). These 16 S sequences were used as a blast database to identify and extract the 16S sequences from all the RoC-ITS reads (*N* = 3489; blastn parameters: blastall v2.2.26 -F F -X 150 -q -5 -r 4 -e 1e-120) [58]. The bioinformatic tool cd-hit-est was used to cluster the 16S portion of the RoC-ITS sequences based on sequence similarity (95% cutoff) at roughly the genus level (v4.8.1; parameters: -T 20 -M 0 -n 12 -c 0.95 -d 5000 -l 20 -g 1) [59]. Clusters with five or more of the 16S sequences (*N* = 3310; 95%) were used to generate clusters of the corresponding RoC-ITS sequences; sequences in small clusters were excluded from further analysis (*N* = 179; 5%). Popular de novo methods for identifying chimera did not prove to be effective on the RoC-ITS sequences and reference sequences were not available for all the organisms in the artificial community. In the absence of vetted reference RoC-ITS sequences, we relied on an iterated clustering process to identify potential chimera under the assumption that most chimera will be distinct and thus poorly represented in a pool of sequences. Therefore, sequences that do not cluster or form only small clusters would be suspect and excluded from the ongoing analysis. Spot checks of these excluded RoC-ITS sequences confirmed that they were chimera.

After removal of chimera, each batch of RoC-ITS sequences was clustered into putative genera were assigned to a taxonomy, multiply aligned, the resulting MSA was improved, corrected, and the ragged ends trimmed. Taxonomic assignment was made by aligning the RoC-ITS sequences to the reference V4 Illumina sequences (see below) using blastn (blastall v2.2.26 -F F -X 150 -q -5 -r 4 -e 1e-120). The taxonomic assignment of the cluster was based on the most abundant taxa assigned to the constituent RoC-ITS sequences; the larger clusters had over 90% of the reads belonging to the same genera. The multiple alignments were carried out using the fast aligner muscle (v3.8.31) [60]. Muscle produced numerous systematic errors including introducing variable gapping patterns in what should be identical portions of the alignment. We developed a script to identify problematic regions and to re-align these shorter segments with a slower but consistent multiple sequence aligner, probcons (v1.1) [56]. The resulting MSA was then iteratively corrected two times to identify and remove infrequent or sporadic errors. The correction process can be described as follows: a given column of the MSA was analyzed: if there was one dominant base at that position (by default dominant meaning that 98% or more of the reads were the same) then the incorrect bases were “corrected” to the dominant base. If there were two or more abundance bases, any low abundance bases present at a frequency of less than 2% were corrected to an N. RoC-ITS sequences with an excess of corrections (*N* > 0.05% * length of the RoC-ITS sequence) were considered outliers and excluded from the cluster and further analysis; manual inspection of these RoC-ITS sequences with large numbers of errors revealed that they were associated with large indels or were chimeric. In the first pass, the number of potential corrections was determined and used to exclude high error rate RoC-ITS sequences, but actual corrections were not made. Corrections were only made during the second iteration thereby preventing the outliers identified in the first round from influencing the final correction step. Beyond identifying these highly erroneous RoC-ITS sequences, correction did not significantly alter the relationship of the sequences, rather resulting in cleaner, easier to interpret phylogenetic trees. Finally, the MSA was trimmed to provide uniform ends and remove any remnants of the RoC-ITS primers; if not removed the variable bases in the primers can result in misleading phylogenetic trees and counts of potential rrns. The precise amount of trimming performed was determined algorithmically to identify the first and last position where 98% or more of the bases in a given column of the MSA were not gaps.

The second round of clustering was designed to remove subtle differences due to chimera and to produce clusters corresponding to individual rrns. The number of informative bases in the MSA that could differentiate between two different rrns was often quite low, so the precise parameters vary depending on the organism involved. To facilitate separation of the distinct rrns, we identified any column in the MSA where the majority base had a frequency of less than 95%. Then sub-sequences involving these variable columns as well as the four neighboring columns were extracted. These sub-sequences were re-clustered with cd-hit-est at a given percent identity between 90 and 99% identity. The resulting individual clusters of sub-sequences containing less than 5% of the input sequences were excluded from further processing as putative chimera; note that an exception was made for the *Pseudomonas* genus where clusters with six or more members were retained to minimize the chance that sequences belonging to the less abundant isolates from this genus were excluded. The remaining clusters were used to generate equivalent clusters using the full-length corrected and trimmed RoC-ITS sequences. These clusters of the full-length RoC-ITS sequences were used to generate consensus sequences which were manually validated and could be used for direct chimera detection. At the 90 percent cutoff, the surviving cluster members were combined, re-aligned with muscle, then the MSA was improved a final time. Following manual inspection of the final MSA to ensure that the trimming and overall alignment looked reasonable, bootstrapped (*N* = 10) phylogenetic trees were generated with RAxML (v8.2.12) [61] and visualized using the Tree of Life (https://itol.embl.de) [62]. The number of distinct clades in the trees could be used to determine whether the clustering percent identity was producing the appropriate number of final clusters; if not, the clustering identity was adjusted upwards until the minimum threshold to produce the correct number of clusters arrived at.

### Statistical tests

Chisq.Test were run as implemented in the Microsoft Excel. Pearson and Spearman correlations were run as implemented in R (version 3.5.1).

### Read error estimates

Error rates were estimated against E. coli K-12 reference 16S sequences. RoC-ITS sub-reads were selected from RoC-ITS reads with 5+ sub-reads and where every sub-read was used. These sub-reads were aligned with blastn (blastall v2.2.26 -F F -e 1e-120) to the 16S portions of the rrns. The best match was assumed to be the correct match. The length of the alignment was the length of the HSP (high scoring pair) plus any unaligned bases from the reference 16S gene. Percent identity was defined as the number of identities divided by the length of the alignment.

### Chimera detection

Chimera detection with vetted reference sequences was performed with the VSearch software package (v.2.14.2) with the vsearch algorithm [53].

### Processing *E. coli* RoC-ITS consensus reads

RoC-ITS sequences derived from *Escherichia coli* K12 were processed just like the artificial community RoC-ITS sequences except that they were not processed through the first, genera-specific, clustering step. Instead, they were pre-filtered to remove highly truncated or artifactual sequences. The pre-filter consisted of a blast search (NCBI blastall v-2.2.26) against the 16S-ITS *E. coli* rrn sequences. Reads that were less than 95 percent identical over the full length of at least one of the 16S-ITS regions (global-identity) were discarded. The remaining steps proceeded as described above for the artificial community.

### Illumina V4 16S libraries

The artificial community sample used here (Table 2) was sequenced both with RoC-ITS and with Illumina sequencing-by-synthesis technology. A barcoded lllumina library was generated via amplification of the V4 hypervariable region of the 16S rRNA gene with the Earth Microbiome modified F515/R806 primers [31] and run on the MiSeq platform along with many other unrelated 16S samples using a MiSeq 600 cycle kit (Illumina, San Diego, CA, USA). After demultiplexing, analysis of the V4 regions was carried out using QIIME2 [30]. Amplicon sequencing variants (ASVs) were generated using the DADA2 subcommand [44] from within QIIME2 release 2018.11 with the parameters “–p-trim-left-f 31 –p-trim-left-r 32 –p-trunc-len-f 220 –p-trunc-len-r 150”. A portion of the Mothur MiSeq SOP [63] was then followed to align reads to the RDP training set v.9 [64] and to remove reads identified as anything other than eubacteria or archaea. Remaining reads were imported back into QIIME2 and chimeras were removed using the “vsearch uchime-denovo” subcommand [53]. ASVs were classified using the “classify-sklearn” command in QIIME2 against release 132 off the Silva SSU database [65].

**Table 2:** Abundance of cd-hit clustered Illumina and RoC-ITS data at various taxonomic levels.

	Phylum	Class	Genus	# of Illumina V4 Reads	# of RoC-ITS Sequences
1	Bacteroidetes	Bacteroidia	Flavobacterium	55,508	981
2	Actinobacteria	Actinobacteria	Micrococcus	38,772	299
3	Proteobacteria	Gammaproteobacteria	Pseudomonas	28,598	492
4	Proteobacteria	Gammaproteobacteria	Pseudomonas	26,951	330
5	Firmicutes	Bacilli	Bacillus	26,521	516
6	Proteobacteria	Gammaproteobacteria	Duganella	12,681	162
7	Proteobacteria	Gammaproteobacteria	Pseudomonas	12,313	143
8	Proteobacteria	Gammaproteobacteria	Pseudomonas	11,697	118
9	Bacteroidetes	Bacteroidia	Mucilaginibacter	9,373	175
10	Proteobacteria	Alphaproteobacteria	Bradyrhizobium	4,135	85
11	Actinobacteria	Actinobacteria	Micrococcus	3,570	11
12	Firmicutes	Bacilli	Bacillus	3,109	68
13	Firmicutes	Bacilli	Bacillus	2,784	62
14	Bacteroidetes	Bacteroidia	Pedobacter	1,723	104
15	Proteobacteria	Gammaproteobacteria	Pseudomonas	1,604	18
16	Proteobacteria	Alphaproteobacteria	Inquilinus	529	1
17	Actinobacteria	Actinobacteria	Rhodococcus	84	1
18	Actinobacteria	Actinobacteria	Mycobacterium	36	2
				Total=	3568

## Results

### *Escherichia coli* K12

**Fig. 2:**
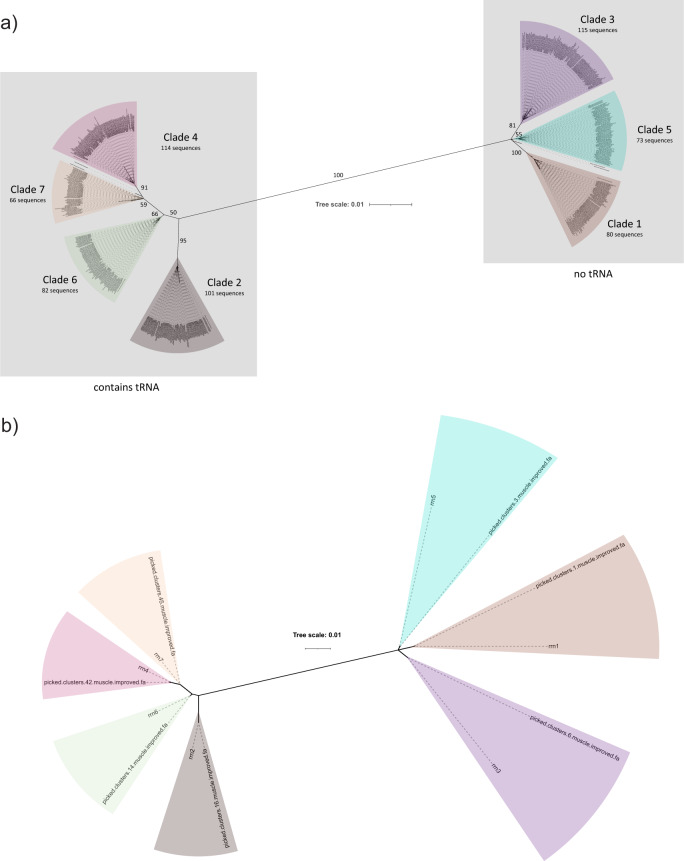
Phylogenetic trees from *E. coli*. The tree scale shows the number of edits per base per unit length. **a** After two rounds of clustering, 628 *E. coli* RoC-ITS sequences (out of 708 initial reads) were combined with the seven *E. coli* K12 reference 16S-ITS sequences, multiply aligned, and converted into an unrooted phylogenetic tree. Bootstrap values are shown for the primary branches. Each of the seven highlighted clades corresponds to one of the reference 16S-ITS sequences, numbered based on their distance from the origin (closest to furthest), along with the number of RoC-ITS sequences associated with each clade. **b** Consensus sequences after clustering RoC-ITS sequences at 99.5% identity. The unrooted phylogenetic tree shows these consensus sequences along with the *E. coli* K12 reference 16S-ITS sequences.

We tested the RoC-ITS approach on a clonal population of *Escherichia coli* K-12. As one of six barcoded samples run on a previously used and cleaned nanopore flow cell, we generated 8000 *E. coli* RoC-ITS reads before the run prematurely terminated. A total of 708 reads were left after filtering and selecting *E. coli*-like reads whose RoC-ITS sequences were derived from five or more iterations around the circular template (i.e., containing 5 or more sub-reads). These RoC-ITS sequences were then processed with the RoC-ITS clustering pipeline at a 90% identity, which removes small clusters, reads with high error rates, and singleton reads, resulting in 622 high-quality RoC-ITS sequences (88%). A phylogenetic tree of these RoC-ITS sequences is organized into seven distinct clusters that correspond to the 7 expected rrns found in the K-12 genome (Fig. 2a). The error rate for sub-reads was calculated to be 98% with a standard deviation of 1%. The error rate of the 708 RoC-ITS sequences was 0.21%; among the 622 good sequences, the error rate was 0.1%. Misalignment due to edge effects may have inflated the error rate; restricting the error analysis to the 16 S portion of the 622 good sequences resulted in an error rate of 0.01%. The ability to isolate clusters whose consensus corresponds to the expected rrns depends on the clustering identity. When clustered at 90% identity, two distinct clusters are resolved, distinguished by the presence or absence of a tRNA in the ITS (Fig. 2a). For the clustering to recapitulate all seven individual rrns, clustering at 99.2% or greater identity was required. The resulting consensus rrns are almost identical to the expected rrns (Fig. 2b) with two of the consensus sequences differing from expectation by a single base substitution. Careful examination of the MSAs associated with these differences reveal that the differences were not due to simple indels, often a source of error in Nanopore reads and frequently associated with polynucleotide stretches, but rather base substitutions with frequencies of 100% among the relevant RoC-ITS sequences. This either points to errors in the reference genome or, more likely, to evolution within the sub-strain used for this experiment.

We examined the number of RoC-ITS sequences associated with each rrn cluster (Supplemental Table 3). Even with random sampling, a statistical test rejected the null model where each rrn cluster should be equally abundant (*χ*^2^ test, *P* = 2.64E-04). The distribution of RoC-ITS sequences has no correlation with the length of the fragments nor is there any reason to think that the primers would work differentially among rrns (all seven should have the same primer annealing sequences). Our current hypothesis is that the compounding effects of amplification and sequencing added more variability to the count of sequences than expected. In the next section, we further test this by comparison with sequences obtained via the Illumina short-read technology. To better understand the contribution of the ITS region to the ability to discern the individual rrns, we examined a tree constructed using the 16S gene alone. This tree had six distinct clades versus the seven seen with the 16S-ITS. By comparing the alignment for the 16S gene alone versus the full 16S-ITS region, we found that 26 versus 301 bases were informative.

### Artificial community

**Fig. 3:**
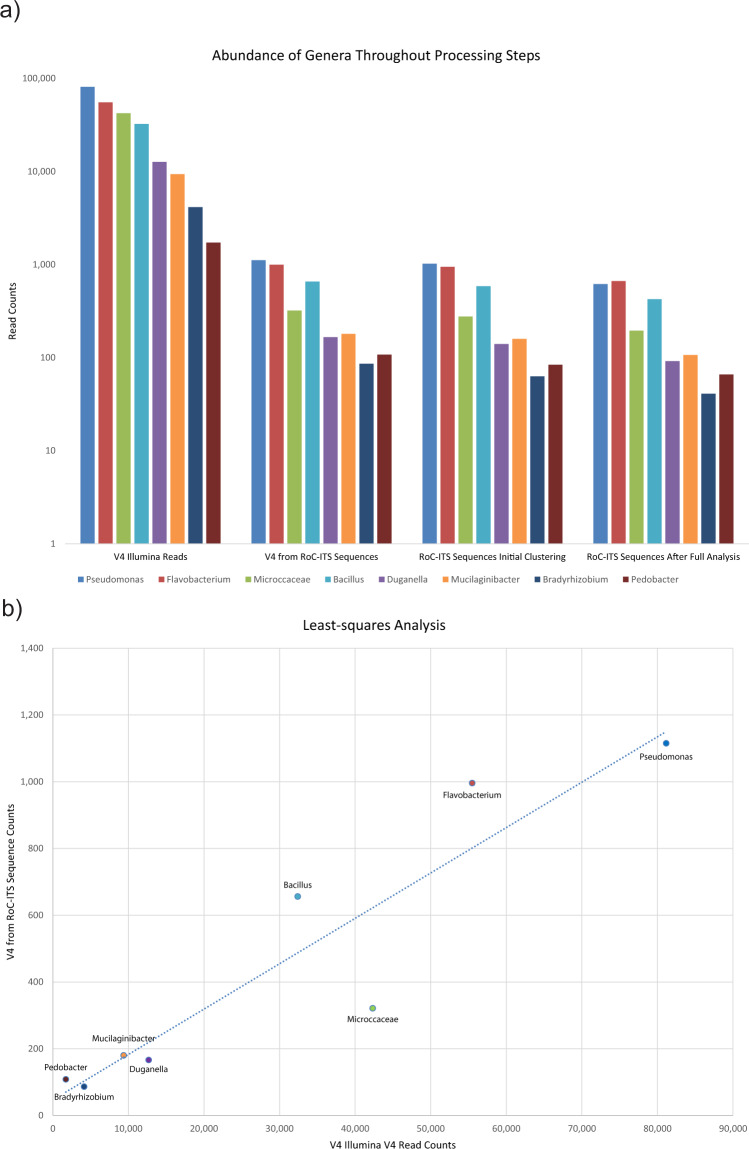
Comparison of genera abundance. **a** Genera during different techniques or at different stages of processing: Illumina reads generated from the V4 region, V4 regions identified on the RoC-ITS sequences, RoC-ITS sequences after the initial clustering to identify genera, and RoC-ITS sequences after being processed fully through the RoC-ITS pipeline to identify high-quality clusters. **b** Least-squares analysis of RoC-ITS and Illumina V4 data with *R*^2^ value of 0.86.

A quantitatively stratified artificial community containing 17 different soil-associated eubacteria was used to test the RoC-ITS approach on more complex samples. This community was constructed from a collection of isolates, some of which represent well-studied organisms, for example *Bacillus subtilis* str. 168, while the majority have yet to be fully sequenced and do not have close relatives with complete genomes in public databases. Some genera in the artificial community were represented by a single isolate, e.g., *Duganella* and *Bacillus*, while other genera had multiple distinct isolates, e.g., *Pseudomonas*. We verified that the primers, both the traditional V4 and the RoC-ITS primers, match the existing reference genomes associated with these genera in the artificial community; when these references have more than one ribosomal operon, we did not find any differences in the primer regions between operons. To establish the makeup of the community by conventional means, we generated 239,988 Illumina 16S V4 amplicon reads on a MiSeq machine (see Methods). The reads were run through the QIIME2 pipeline, which identified 18 different Amplicon Sequence Variants (ASVs) belonging to 11 different genera (Table 3). At the same time, the RoC-ITS library was prepared from the artificial community and sequenced on a previously used (spent) Nanopore flow cell to generate 31,215 Nanopore reads, of which 25,489 passed the initial Nanopore quality filters. The Nanopore reads were then processed through the RoC-ITS consensus pipeline to generate 3667 high-quality RoC-ITS sequences (see Methods). Briefly, only Nanopore reads with consistent barcodes, individual inserts of the expected length between 1500 and 3500 bp, and with 5 or more sub-reads, were kept and used to generate RoC-ITS sequences that were used for comparison with the Illumina data and for independent phylogenetic analysis. Overall, the quantification is similar between the V4 and RoC-ITS sequences (Table 3; Fig. 3) with significant correlation (Pearson: *r*  =  0.93, *P* = 8.209 × 10^−4^; Spearman: *r*  =  0.929, *P* = 2.232 × 10^−3^). Among the more abundant microbes, the *Microccaceae* are less abundant in the RoC-ITS data than expected. There are smaller discrepancies in the less abundant organisms as well. While such differences are not unexpected in PCR studies, it is also possible that these reflect subtle mismatches in the amplification primers used to isolate one or more of the 16S-ITS regions. Given the absence of explicit reference genomes, it is impossible to disregard the possibility at this time. The differences do not seem to correlate with the size of the 16S-ITS region suggesting length bias is not an important factor.

**Table 3:** Abundance of genera at different stages of the RoC-ITS processing.

Genera	V4 Illumina Reads	V4 from RoC-ITS Sequences	Expected RoC-ITS #	RoC-ITS Sequences Initial Clustering	RoC-ITS Sequences After Full Analysis	ROC-ITS Rank	V4 Rank	Number of Genera Clusters	Largest Cluster	Largest Cluster as Percent	Surviving Processing
Pseudomonas	81,163	1115	1230	1022	618	1	1	14	753	73.7%	60.5%
Flavobacterium	55,508	996	841	944	666	2	2	11	806	85.4%	70.6%
Microccaceae	42,342	321	642	277	195	4	3	4	235	84.8%	70.4%
Bacillus	32,414	656	491	585	425	3	4	8	472	80.7%	72.6%
Duganella	12,681	166	192	140	92	6	5	2	134	95.7%	65.7%
Mucilaginibacter	9373	180	142	159	107	5	6	5	107	67.3%	67.3%
Bradyrhizobium	4135	86	63	63	41	8	7	3	50	79.4%	65.1%
Pedobacter	1723	108	26	84	66	7	8	1	84	100.0%	78.6%
**Total**	239,339	3628	3628	2828	**2210**						

Additionally, genera data is ranked, related to the cd-hit clusters, and processing survival metrics are presented.

### Phylogenetic analysis and classification of the artificial community

To further characterize the artificial community, we developed an approach for analyzing the RoC-ITS sequences that do not depend on knowing the composition of the community in advance. First, we organized the sequences roughly by genus. To accomplish this, we excluded the poorly characterized ITS portion of the RoC-ITS sequences by extracting the 16S portion of each sequence using a combination of the established 16S primers and sequence similarity (see methods). We were able to isolate the intact 16S gene in 3628 of the 3667 RoC-ITS sequences. Of these, 3568 could be assigned to a particular genus based on BLAST using the QIIME V4 consensus sequences (Table 2). These 16S segments were then clustered with cd-hit-est at 95% identity and produced 44 clusters containing five or more 16S sequences per cluster; these 44 clusters accounted for 3584 of the RoC-ITS reads. These clusters were initially classified by the majority genus present in the cluster. The full-length RoC-ITS sequences were grouped into eight genera-specific clusters and then examined individually in more detail (Table 3). Note that the least abundant genera in the artificial community (*Inquilinus*, *Rhodococcus*, and *Mycobacterium*) were all intentionally excluded by this process and did not have sufficient read counts to provide for a robust analysis.

### Bacillus subtilis

**Fig. 4:**
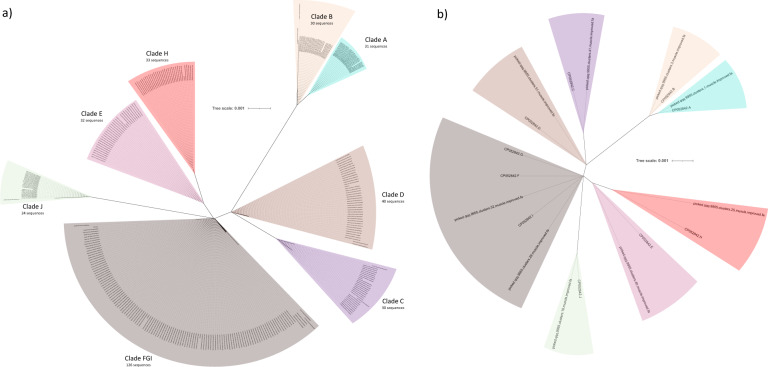
Phylogenetic analysis of the *Bacillus* subtilis RoC-ITS sequences. The tree scale shows the number of edits per base per unit length. **a** Phylogenetic tree of *Bacillus* RoC-ITS sequences along with the 10 reference *Bacillus subtilus* str 168 rrns (labeled CP052842.A-J with the letters corresponding to their order in ascending base pair coordinates on the reference genome). The RoC-ITS reads are found in eight distinct clades in accordance with the reference rrns. The single largest clade containing three reference rrns (F, G, and I) and consists of the two identical rrns plus a third rrn that differs by only two deleted bases. **b** A phylogenetic tree of the reference rrns and the consensus sequences derived from the nine clusters produced after clustering the *Bacillus* RoC-ITS sequences at 99.55% identity.

The best-characterized member of the artificial community was *Bacillus subtilis* str. 168. The reference genome contains ten rrns, two of which are identical. Several of the distinct rrns differ from each other by only one or two base pairs. A total of 585 RoC-ITS sequences were identified as related to *Bacillus*. Processing of the RoC-ITS sequences with a 90% clustering threshold removed low quality and suspected chimeric sequences leaving 425 high quality RoC-ITS sequences. A phylogenetic tree derived from the resulting sequences and the analogous 16S-ITS sequences extracted from the *Bacillus subtilis* str. 168 reference genome shows that RoC-ITS sequences matched *a priori* expectations, with eight major clades clearly identifiable (Fig. 4a). Clustering at much higher identity (99.55%) is required to isolate clusters that correspond to the 9 distinct rrns known to occur though also reducing the total number of usable RoC-ITS sequences to 336. The reason that there were eight distinct clades in the tree but that nine distinct clusters of sequences could be isolated was due to the nature of the tree construction algorithm. One of the clusters differs only due to the deletion of a single base pair and gapped positions were ignored during tree construction. The consensus sequences derived from multiple alignment of these RoC-ITS sequence clusters faithfully recapitulated the reference sequences from *Bacillus subtilis* str. 168 rrns (Fig. 4b). The number of reads associated with each cluster varied between 23 and 41 (Supplementary Table 4). Unlike the *E. coli* example reported earlier, the variability in reads per rrn observed here was consistent with random sampling (χ^2^ test, *P* = 0.14). When constructed from the 16S gene alone, the resulting tree had seven distinct clades versus the eight seen with the 16S-ITS. By examining the alignment for the 16S gene alone versus the full 16S-ITS region, we found that 9 versus 200 bases were informative. In the context of the artificial community, where *Bacillus subtilis* was represented by a single well-characterized strain, our results, therefore, demonstrate that we can both qualitatively and quantitatively assess the abundance of a genome and its individual rrns.

### Duganella

**Fig. 5:**
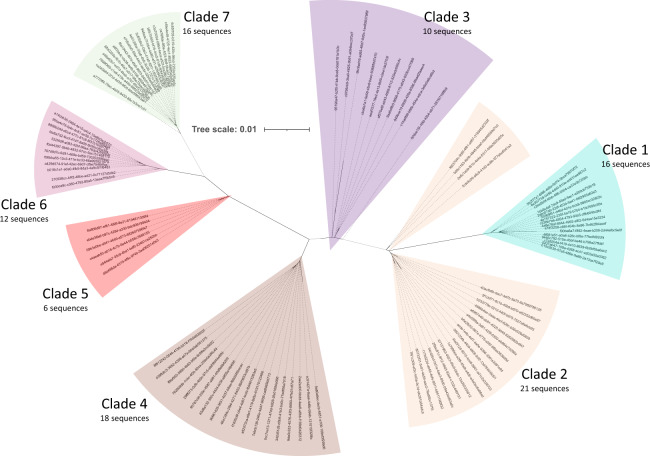
*Duganella* phylogenetic tree based on clustering at 90% identity. The 99 *Duganella* RoC-ITS sequences form 7 distinct clades consistent with the number of rrns in other related *Duganella* strains with complete genomes. Note that because many of the differences were associated with gapped regions, for this tree all dashes in the MSA were replaced with A’s thereby better capturing the relationship between the distinct rrns. The tree scale shows the number of edits per base per unit length.

Analysis of the *Duganella* RoC-ITS sequences indicated that the isolate present in the artificial community is not a close match to any of the published *Duganella* complete or draft genomes, with the best 16S matches having ~97% identity. The complete *Duganella* genomes that we examined all had seven distinct rrns. Clustering and analysis of the Duganella RoC-ITS reads also produced seven distinct clades at both 90% (Fig. 5) and 99% clustering cutoffs. Our tests indicate that the number of reads per rrn are not significantly different from a random distribution (*χ*^2^ test, *P* = 0.09; Supplemental Table 5). The 16S portion by itself has sufficient information to recreate this tree structure, but only 8 bases provide informative sites (versus 108 in the full 16S-ITS region).

### Other genera

We analyzed all the genera found in the artificial community by processing the RoC-ITS sequences through our analysis pipeline and generating trees from the resulting MSAs. While these data were consistent with existing reference genome data, the absence of closely related reference genomes, and variability (or lack thereof) in the number of rrns associated with these genera made statistical analysis of little value. For example, both the *Bradyrhozobium* and *Mucilinobacter* genera are thought to have one or at most two identical rrns. Our results are consistent with there being a single distinct rrn sequence (Supplemental Figs. 3 and 4). The *Pseudomonas* genera were comprised of four distinct isolates but there is considerable variability in the number of rrns in related reference genomes. The *Pseudomonas* RoC-ITS sequences, when used to construct a phylogenetic tree, roughly clustered into clades that corresponded to the four most abundant Illumina ASVs identified by QIME (Supplementary Fig. 5) with the same corresponding rank abundances. The *Flavobacterium*, *Micrococcus*, and *Pedobacter* genera indicate that they possess 6, 5, and 3 distinct rrns though the *Flavobacterium* data suggests that one of the rrns is highly duplicated (*n* = 4) bringing its total number of predicted rrns to 9 (Supplemental Figs. 6–8). All these values are reasonable predictions given available reference genomes but the specific values are currently unverifiable.

Furthermore, having identified all the prospective clusters of RoC-ITS sequences, we produced prospective consensus sequences that were then used to perform chimera detection using vsearch toolkit. Using these vetted reference sequences, we were able to identify and remove 98% of the suspected chimera in a fraction of the time than that required by the clustering-based approach that we relied on in the absence of good references.

## Discussion

As demonstrated here, increased resolution through the inclusion of the less-constrained ITS region can greatly enhance compositional analysis of microbial communities. This allows for strain-level identification, which could be used to correlate subtle shifts in community composition with environmental factors or experimental outcomes. By examining the utility of long-read data to identify individual rrns, this method extends work by other researchers who have used long-read sequencing strategies to examine the entire ribosomal RNA to obtain strain level identifications [32, 66]. The UMI-PCR-based approach advocated in these papers has the advantage of simplicity, but it does require a bottlenecking step such that the population of starting molecules is considerably smaller than the sequencing depth. While reducing the effective depth of sequencing, this results in each UMI being sequenced multiple times resulting in a consensus sequence with a low potential for error. While RoC-ITS can be enhanced with bottlenecking, this approach was not employed here and is not essential as each sufficiently long read confirms itself and provides actionable information. Instead, the UMIs were used to identify and avoid double-counting potential PCR-induced over-amplification; however, this did not appear to be a serious issue in these circumstances with only a limited depth of sequencing. Furthermore, the ability to discern individual rrns within organisms means that for many species, multiple independent markers can be used to assess and correlate quantification between the markers, thereby providing increased accuracy and sensitivity. Note that this increased sensitivity comes at the price of increased complexity, especially when the number of rrns is not known in advance and the tools to make use of this information do not exist.

Analysis of the 16S gene alone shows that individual rrns can, generally, be identified but that there is a much greater information content (roughly tenfold more) in the ITS region. Note that some of these differences, for example in *Bacillus* and *Escherichia*, are due to the insertion of entire tRNA genes which could be viewed as a singular event. While the full rrn can be sequenced [32] and doubtless contains additional markers for distinguishing individual rrns and strains of microbes, it is not clear whether this would provide sufficient benefit to justify the potential noise introduced with long PCR especially when applied to complex and potentially poorly characterized microbial communities.

The work outlined here only demonstrates that such an approach would be feasible, as real-world cases would likely be far more complex, possibly with more subtle strain-level variation. Fortunately, existing tools, such as dbOTU3 [67], are well suited to this role of “grouping” sequences that are highly correlated across large numbers of different samples. The explicit knowledge of these markers could be used to target particular strains for isolation and genome sequencing to better understand the specific genomic differences that underly these population shifts. In turn, this could lead to better functional characterization of the underlying genes and their roles in a community. Ultimately, a holistic approach may produce more robust models describing how microbial communities function and respond to other microbes and to changes in the environment.

We demonstrated that the inclusion of the ITS region can provide valuable resolving power to interrogate microbial communities; however, many technical challenges remain. The stringent criteria used here to retain reads for analysis meant that 80–90% of the Nanopore reads were left unused. The majority of these reads were abandoned because they were too short and therefore did not generate the minimum number of sub-reads (*N* = 5) that we required, so improvements to the production of longer rolling-circle products would be a boon. There are already products that select longer DNA fragments, 10 kb or larger, that might work with minor changes to the protocols described herein. Furthermore, improvements to the Nanopore flow cells and base-calling software could also improve the reliability of the read data, allowing the shorter reads to be useful. For example, multiple sequence alignment is key to organizing and structuring RoC-ITS sequences into clusters. Integrating the depth of coverage supporting any given RoC-ITS sequence to provide a quality score could, with the development of new software, be used to produce better error correction and consensus calling. Alternative sequencing approaches, such as the PacBio platform, can automatically iterate over the template to produce higher-quality reads [32, 68]. Clever Illumina library construction strategies (such as that used by LoopSeq) can allow sequencing of the entire 16 S gene and may be amenable to sequencing of the ITS and 16S gene [44]. Given the relatively high cost of single-molecule sequencing technologies, it may be more cost-effective to invest in cataloging the ITS diversity so that in the future, given a sufficiently rich database of ITS sequences with clear taxonomic assignments, reliance on the 16S may be pared down or eliminated altogether allowing high-resolution taxonomic assignment through the use of Illumina-based protocols focused on the ITS region alone.

A significant number of reads were also lost due to template swapping that likely occurred during the PCR steps, resulting in chimeric reads. It is unclear if this problem is exasperated by the length of the template, the high conservation of the template, or is just more apparent due to specifics of the experiment performed with discrete isolates. Many of the chimera are easily detected because they were the result of template jumping to or from the most abundant organisms, *Flavobacteria* and *Pseudomonades*, and the fact these jumps do not seem to be restricted to the conserved 16S portion of the reads. Undoubtedly some of the noise associated with the sequence analysis was also due to the formation of within-genus chimera, which can be much harder to detect. Fine-tuning the molecular biology may be able to minimize this effect, but it will likely remain a problem. Developing a much more comprehensive database of rrns in the future would allow the use of existing fast tools for identifying and excluding chimeras, even within genera.

While there are large databases of 16S and 16S variable regions already available, these are relatively low-resolution databases that do not come close to capturing the full richness of the microbial world. Increasing resolution using highly variable segments like the eubacterial ITS region makes it feasible to start to track strains even in complex communities. High-throughput single-molecule techniques make this a possibility, and our work highlights the power of applying such techniques to microbial populations. Further software development will likely be needed to deal with larger volumes of data and to organize it into distinct taxonomic groups in the absence of prior information. Our current approach relies on the strategic use of computationally expensive approaches like MSA to ensure a high confidence consensus RoC-ITS sequence. In particular, we use MSA to generate consensus sequences either during the production of a RoC-ITS sequence from its underlying sub-reads or when we generate a consensus from a set of RoC-ITS sequences representing a distinct 16S-ITS operon. In the first case, the number of sub-reads that contributes to a RoC-ITS sequence is inherently limited by the technology and all the sequences should be nearly identical limiting the computational burden. In the second instance, the number of sequences is somewhat unbounded and given a larger dataset this could prove computationally problematic. In these cases, as these are already highly similar sequences, we would recommend capping the number of sequences used to generate the RoC-ITS consensus sequence to limit the computational complexity of the alignment. The overall clustering steps rely on much faster k-mer-based techniques such as those implemented in the cd-hit package. However, with more comprehensive 16S-ITS databases, even more rapid approaches relying on k-mers may be able to assign sub-reads to known reference sequences and establish a probability that the sub-reads all are derived from a known 16S-ITS reference. Then, the more expensive MSA-based steps would only be needed when no 16S-ITS reference could be discovered. There are other refinements or improvements that might follow directly from this work; we did not check whether the primers used here would work universally in Archaea, but since universal 16S primers already exist, it’s likely that similarly universal primers can be identified in the 23S gene. By using a wholly different pair of primers, this technique could be applied to the ITS regions from fungal organisms or used to characterize large loci from any organism. Overall, there is much promise for high-throughput, high-resolution approaches that can elucidate microbial composition and correlate composition with functional and environmental variables.

## Supplementary information

Legends

Table S1

Table S2

Table S3

Table S4

Table S5

Figure S1

Figure S2

Figure S3

Figure S4

Figure S5

Figure S6

Figure S7

Figure S8

## Data Availability

Code, walk-throughs, example data, and the MSA for the genera discussed in the paper are available on GitHub at: https://github.com/mondegreen/RoC-ITS2.

## Data availability

The raw Nanopore sequencing data in fastq format as well as the V4 Illumina MiSeq paired-end reads have been submitted to the NCBI Sequence Read Archive under project PRJNA669399.

## References

[1] Srivastava AK, Schlessinger D (1990). Mechanism and regulation of bacterial ribosomal RNA processing. Annu Rev Microbiol.

[2] Brewer TE, Albertsen M, Edwards A, Kirkegaard RH, Rocha EPC, Fierer N (2020). Unlinked rRNA genes are widespread among bacteria and archaea. ISME J.

[3] Apirion D, Miczak A (1993). RNA processing in prokaryotic cells. Bioessays.

[4] Espejo RT, Plaza N (2018). Multiple ribosomal RNA operons in bacteria; their concerted evolution and potential consequences on the rate of evolution of their 16S rRNA. Front Microbiol.

[5] Roller BRK, Stoddard SF, Schmidt TM (2016). Exploiting rRNA operon copy number to investigate bacterial reproductive strategies. Nat Microbiol.

[6] Lim K, Furuta Y, Kobayashi I (2012). Large variations in bacterial ribosomal RNA Genes. Mol Biol Evol.

[7] Ludwig W, Strunk O, Klugbauer S, Klugbauer N, Weizenegger M, Neumaier J (1998). Bacterial phylogeny based on comparative sequence analysis. Electrophoresis.

[8] Woese CR, Fox GE (1977). Phylogenetic structure of the prokaryotic domain: the primary kingdoms. Proc Natl Acad Sci.

[9] Lane DJ, Pace B, Olsen GJ, Stahl DA, Sogin ML, Pace NR (1985). Rapid determination of 16S ribosomal RNA sequences for phylogenetic analyses. Proc Natl Acad Sci.

[10] Park YH, Hori H, Suzuki K, Osawa S, Komagata K (1987). Phylogenetic analysis of the coryneform bacteria by 5S rRNA sequences. J Bacteriol.

[11] Szymanski M, Barciszewska MZ, Erdmann VA, Barciszewski J (2002). 5S Ribosomal RNA Database. Nucleic Acids Res.

[12] Pace NR (2018). The small things can matter. PLoS Biol.

[13] Gürtler V (1999). The role of recombination and mutation in 16S–23S rDNA spacer rearrangements. Gene.

[14] Snyder AK, Adkins KZ, Rio RVM (2011). Use of the internal transcribed spacer (ITS) regions to examine symbiont divergence and as a diagnostic tool for sodalis-related bacteria. Insects.

[15] Man SM, Kaakoush NO, Octavia S, Mitchell H (2010). The internal transcribed spacer region, a new tool for use in species differentiation and delineation of systematic relationships within the Campylobacter genus. Appl Environ Microbiol.

[16] Liguori AP, Warrington SD, Ginther JL, Pearson T, Bowers J, Glass MB (2011). Diversity of 16S-23S rDNA Internal Transcribed Spacer (ITS) reveals phylogenetic relationships in Burkholderia pseudomallei and its near-neighbors. PLoS One.

[17] Boyer SL, Flechtner VR, Johansen JR (2001). Is the 16S–23S rRNA internal transcribed spacer region a good tool for use in molecular systematics and population genetics? A case study in cyanobacteria. Mol Biol Evol.

[18] Fisher MM, Triplett EW (1999). Automated approach for ribosomal intergenic spacer analysis of microbial diversity and its application to freshwater bacterial communities. Appl Environ Microbiol.

[19] Brown BL, Watson M, Minot SS, Rivera MC, Franklin RB (2017). MinION^TM^ nanopore sequencing of environmental metagenomes: a synthetic approach. Gigascience.

[20] Hernando-Morales V, Varela MM, Needham DM, Cram J, Fuhrman JA, Teira E (2018). Vertical and seasonal patterns control bacterioplankton communities at two horizontally coherent coastal upwelling sites off Galicia (NW Spain). Microb Ecol.

[21] Johnson JS, Spakowicz DJ, Hong B-Y, Petersen LM, Demkowicz P, Chen L (2019). Evaluation of 16S rRNA gene sequencing for species and strain-level microbiome analysis. Nat Commun.

[22] Sogin ML, Morrison HG, Huber JA, Mark Welch D, Huse SM, Neal PR (2006). Microbial diversity in the deep sea and the underexplored ‘rare biosphere’. Proc Natl Acad Sci USA.

[23] Nossa CW, Oberdorf WE, Yang L, Aas JA, Paster BJ, Desantis TZ (2010). Design of 16S rRNA gene primers for 454 pyrosequencing of the human foregut microbiome. World J Gastroenterol.

[24] Bentley DR, Balasubramanian S, Swerdlow HP, Smith GP, Milton J, Brown CG (2008). Accurate whole human genome sequencing using reversible terminator chemistry. Nature.

[25] Thompson LR, Sanders JG, McDonald D, Amir A, Ladau J, Locey KJ (2017). A communal catalogue reveals Earth’s multiscale microbial diversity. Nature.

[26] Kapustina Ž, Medžiūnė J, Alzbutas G, Rokaitis I, Matjošaitis K, Mackevičius G (2021). High-resolution microbiome analysis enabled by linking of 16S rRNA gene sequences with adjacent genomic contexts. Microb Genom.

[27] Tyson GW, Chapman J, Hugenholtz P, Allen EE, Ram RJ, Richardson PM (2004). Community structure and metabolism through reconstruction of microbial genomes from the environment. Nature.

[28] Venter JC, Remington K, Heidelberg JF, Halpern AL, Rusch D, Eisen JA (2004). Environmental genome shotgun sequencing of the Sargasso Sea. Science.

[29] Rusch DB, Halpern AL, Sutton G, Heidelberg KB, Williamson S, Yooseph S (2007). The Sorcerer II Global Ocean Sampling expedition: northwest Atlantic through eastern tropical Pacific. PLoS Biol.

[30] Bolyen E, Rideout JR, Dillon MR, Bokulich NA, Abnet CC, Al-Ghalith GA (2019). Reproducible, interactive, scalable and extensible microbiome data science using QIIME 2. Nat Biotechnol.

[31] Caporaso JG, Kuczynski J, Stombaugh J, Bittinger K, Bushman FD, Costello EK (2010). QIIME allows analysis of high-throughput community sequencing data. Nat Methods.

[32] Karst SM, Ziels RM, Kirkegaard RH, Sørensen EA, McDonald D, Zhu Q (2021). High-accuracy long-read amplicon sequences using unique molecular identifiers with Nanopore or PacBio sequencing. Nat Methods.

[33] Jamy M, Foster R, Barbera P, Czech L, Kozlov A, Stamatakis A (2020). Long-read metabarcoding of the eukaryotic rDNA operon to phylogenetically and taxonomically resolve environmental diversity. Mol Ecol Resour.

[34] Leggett RM, Clark MD (2017). A world of opportunities with nanopore sequencing. J Exp Bot.

[35] Jain M, Olsen HE, Paten B, Akeson M (2016). The Oxford nanopore MinION: delivery of nanopore sequencing to the genomics community. Genome Biol.

[36] Eid J, Fehr A, Gray J, Luong K, Lyle J, Otto G (2009). Real-time DNA sequencing from single polymerase molecules. Science.

[37] Graf J, Ledala N, Caimano MJ, Jackson E, Gratalo D, Fasulo D (2021). High-resolution differentiation of enteric bacteria in premature infant fecal microbiomes using a novel rRNA amplicon. mBio.

[38] Martijn J, Lind AE, Schön ME, Spiertz I, Juzokaite L, Bunikis I (2019). Confident phylogenetic identification of uncultured prokaryotes through long read amplicon sequencing of the 16S-ITS-23S rRNA operon. Environ Microbiol.

[39] Okazaki Y, Fujinaga S, Salcher MM, Callieri C, Tanaka A, Kohzu A (2021). Microdiversity and phylogeographic diversification of bacterioplankton in pelagic freshwater systems revealed through long-read amplicon sequencing. Microbiome.

[40] Miga KH, Koren S, Rhie A, Vollger MR, Gershman A, Bzikadze A (2020). Telomere-to-telomere assembly of a complete human X chromosome. Nature.

[41] Wenger AM, Peluso P, Rowell WJ, Chang P-C, Hall RJ, Concepcion GT (2019). Accurate circular consensus long-read sequencing improves variant detection and assembly of a human genome. Nat Biotechnol.

[42] Matsuo Y, Komiya S, Yasumizu Y, Yasuoka Y, Mizushima K, Takagi T (2021). Full-length 16S rRNA gene amplicon analysis of human gut microbiota using MinIONTM nanopore sequencing confers species-level resolution. BMC Microbiol.

[43] Calus ST, Ijaz UZ, Pinto AJ (2018). NanoAmpli-Seq: a workflow for amplicon sequencing for mixed microbial communities on the nanopore sequencing platform. Gigascience.

[44] Callahan BJ, Wong J, Heiner C, Oh S, Theriot CM, Gulati AS (2019). High-throughput amplicon sequencing of the full-length 16S rRNA gene with single-nucleotide resolution. Nucleic Acids Res.

[45] Benítez-Páez A, Portune KJ, Sanz Y (2016). Species-level resolution of 16S rRNA gene amplicons sequenced through the MinION^TM^ portable nanopore sequencer. Gigascience.

[46] Kumar V, Vollbrecht T, Chernyshev M, Mohan S, Hanst B, Bavafa N (2019). Long-read amplicon denoising. Nucleic Acids Res.

[47] Wick RR, Judd LM, Holt KE (2019). Performance of neural network basecalling tools for Oxford Nanopore sequencing. Genome Biol.

[48] Lane D. 16S/23S rRNA sequencing. In: Stackebrandt E, Goodfellow M (eds). *Nucleic acid techniques in bacterial systematics*. 1991. Wiley, New York, pp 115–75.

[49] Miller CS, Handley KM, Wrighton KC, Frischkorn KR, Thomas BC, Banfield JF (2013). Short-read assembly of full-length 16S amplicons reveals bacterial diversity in subsurface sediments. PLoS One.

[50] Hunt DE, Klepac-Ceraj V, Acinas SG, Gautier C, Bertilsson S, Polz MF (2006). Evaluation of 23S rRNA PCR primers for use in phylogenetic studies of bacterial diversity. Appl Environ Microbiol.

[51] Volden R, Palmer T, Byrne A, Cole C, Schmitz RJ, Green RE (2018). Improving nanopore read accuracy with the R2C2 method enables the sequencing of highly multiplexed full-length single-cell cDNA. Proc Natl Acad Sci.

[52] Gibson DG, Young L, Chuang R-Y, Venter JC, Hutchison CA, Smith HO (2009). Enzymatic assembly of DNA molecules up to several hundred kilobases. Nat Methods.

[53] Rognes T, Flouri T, Nichols B, Quince C, Mahé F (2016). VSEARCH: a versatile open source tool for metagenomics. PeerJ.

[54] Eddy SR (2009). A new generation of homology search tools based on probabilistic inference. Genome Inform.

[55] Morisse P, Marchet C, Limasset A, Lecroq T, Lefebvre A (2021). Scalable long read self-correction and assembly polishing with multiple sequence alignment. Sci Rep.

[56] Do CB, Mahabhashyam MSP, Brudno M, Batzoglou S (2005). ProbCons: Probabilistic consistency-based multiple sequence alignment. Genome Res.

[57] dos Santos HRM, Argolo CS, Argôlo-Filho RC, Loguercio LL (2019). A 16S rDNA PCR-based theoretical to actual delta approach on culturable mock communities revealed severe losses of diversity information. BMC Microbiol.

[58] Altschul SF, Gish W, Miller W, Myers EW, Lipman DJ (1990). Basic local alignment search tool. J Mol Biol.

[59] Fu L, Niu B, Zhu Z, Wu S, Li W (2012). CD-HIT: accelerated for clustering the next-generation sequencing data. Bioinformatics.

[60] Edgar RC (2004). MUSCLE: multiple sequence alignment with high accuracy and high throughput. Nucleic Acids Res.

[61] Stamatakis A (2014). RAxML version 8: a tool for phylogenetic analysis and post-analysis of large phylogenies. Bioinformatics.

[62] Letunic I, Bork P (2019). Interactive Tree Of Life (iTOL) v4: recent updates and new developments. Nucleic Acids Res.

[63] Kozich JJ, Westcott SL, Baxter NT, Highlander SK, Schloss PD (2013). Development of a dual-index sequencing strategy and curation pipeline for analyzing amplicon sequence data on the MiSeq illumina sequencing platform. Appl Environ Microbiol.

[64] Cole JR, Wang Q, Fish JA, Chai B, McGarrell DM, Sun Y (2014). Ribosomal Database Project: data and tools for high throughput rRNA analysis. Nucleic Acids Res.

[65] Quast C, Pruesse E, Yilmaz P, Gerken J, Schweer T, Yarza P (2013). The SILVA ribosomal RNA gene database project: improved data processing and web-based tools. Nucleic Acids Res.

[66] de Oliveira Martins L, Page AJ, Mather AE, Charles IG (2019). Taxonomic resolution of the ribosomal RNA operon in bacteria: implications for its use with long-read sequencing. NAR Genom Bioinform.

[67] Olesen SW, Duvallet C, Alm EJ (2017). dbOTU3: a new implementation of distribution-based OTU calling. PLoS One.

[68] Fichot EB, Norman RS (2013). Microbial phylogenetic profiling with the Pacific Biosciences sequencing platform. Microbiome.

