# Supplementary material for: High-resolution phylogenetic and population genetic analysis of microbial communities with RoC-ITS
Source: ISME Commun. 2022 Oct 10;2:99. doi: 10.1038/s43705-022-00183-8 (PMC9723582; doi:10.1038/s43705-022-00183-8)
Supplement: Supplementary file 1 — Legends [file 43705_2022_183_MOESM1_ESM.pdf]

Supplemental Figure 1. Length distribution of Nanopore reads from artificial community.

Supplemental Figure 2. Length distribution of sub-reads derived from artificial community.

Supplemental Figure 3. Phylogenetic tree of *Bradyrhozobium* RoC-ITS sequences. Tree is consistent with a single ribosomal operon or multiple identical ribosomal operons. The tree scale shows the number of edits per base per unit length.

Supplemental Figure 4. Phylogenetic tree of *Mucilinobacter* RoC-ITS sequences. Tree is consistent with a single ribosomal operon or multiple identical ribosomal operons. The tree scale shows the number of edits per base per unit length.

Supplemental Figure 5. *Pseudomonas* RoC-ITS sequences phylogenetic tree. The four most abundant ASV as identified by the Illumina V4 sequences (labeled 1 through 4 in order from most abundant to least) highlighted with distinctly colored wedges correspond to the four distinct clades of RoC-ITS sequences. Each wedge presumably represents a distinct species of Pseudomonas present in the artificial community. The tree scale shows the number of edits per base per unit length.

Supplemental Figure 6. Phylogenetic tree of *Flavobacterium* RoC-ITS sequences. Tree is consistent there being 6 distinct ribosomal operons and 9 ribosomal operons in total. The tree scale shows the number of edits per base per unit length.

Supplemental Figure 7. Phylogenetic tree of *Micrococcus* RoC-ITS sequences. Tree is consistent there being 5 ribosomal operons. The tree scale shows the number of edits per base per unit length.

Supplemental Figure 8. *Pedobacter* Phylogenetic tree. Tree is consistent with three ribosomal operons. The tree scale shows the number of edits per base per unit length.

Supplemental Table 1. 16S-ITS PCR Program: PCR program to amplify 16S-ITS region.

Supplemental Table 1. Splint PCR Program: PCR program to amplify splint DNA.

Supplemental Table 3. Chi-square test for *E. coli*: Chi-square test whether there is a statistically significant difference between ITS specific read abundance and random expectation for *Escherichia coli*.

Supplemental Table 4. Chi-square test for *B. subtilus*: Chi-square test whether there is a statistically significant difference between ITS specific read abundance and random expectation for *Bacillus subtilus*.

Supplemental Table 5. Chi-square test for *Duganella*: Chi-square test whether there is a statistically significant difference between ITS specific read abundance and random expectation for *Duganella*.
