# Supplementary material for: High-resolution phylogenetic and population genetic analysis of microbial communities with RoC-ITS
Source: ISME Commun. 2022 Oct 10;2:99. doi: 10.1038/s43705-022-00183-8 (PMC9723582; doi:10.1038/s43705-022-00183-8)
Supplement: Supplementary file 2 — Table S1 [file 43705_2022_183_MOESM2_ESM.pdf]

Table S1

Segment	Temp (C)	Time (sec)	Cycles	Primers
Initial denaturation	95	3:00	1	RoC-ITS 27F 16S only
Annealing	46	0:20		
Extension	72	5:00		
Cleanup				
Denaturation	95	3:00	1	RoC-ITS 189r 23S only
Annealing	46	0:20		
Extension	72	5:00		
Cleanup				
Initial denaturation	95	3:00	1	RoC-ITS 27F Unique + RoC-ITS 189r Unique
Denaturation	98	0:20	12	
Annealing	60	0:20		
Extension	72	5:00		
Final extension	72	10:00	1	
Hold	4	0:00	1	
Cleanup				
