# Supplementary material for: High-resolution phylogenetic and population genetic analysis of microbial communities with RoC-ITS
Source: ISME Commun. 2022 Oct 10;2:99. doi: 10.1038/s43705-022-00183-8 (PMC9723582; doi:10.1038/s43705-022-00183-8)
Supplement: Supplementary file 3 — Table S2 [file 43705_2022_183_MOESM3_ESM.pdf]

Table S2

Segment	Temp (C)	Time (sec)	Cycles
Initial denaturation	95	3:00	1
Denaturation	98	0:20	25
Annealing	60	0:15	
Extension	72	0:30	
Final extension	72	10:00	1
Hold	4	0:00	1
