# Supplementary material for: High-resolution phylogenetic and population genetic analysis of microbial communities with RoC-ITS
Source: ISME Commun. 2022 Oct 10;2:99. doi: 10.1038/s43705-022-00183-8 (PMC9723582; doi:10.1038/s43705-022-00183-8)
Supplement: Supplementary file 4 — Table S3 [file 43705_2022_183_MOESM4_ESM.pdf]

Table S3

	Ecoli	Expected %	Expected #
rrn1	94	0.142857	90
rrn2	82	0.142857	90
rrn3	114	0.142857	90
rrn4	70	0.142857	90
rrn5	70	0.142857	90
rrn6	82	0.142857	90
rrn7	118	0.142857	90
**Total**	630		
**Chi-squared p-value**	0.000264		
