# Supplementary material for: High-resolution phylogenetic and population genetic analysis of microbial communities with RoC-ITS
Source: ISME Commun. 2022 Oct 10;2:99. doi: 10.1038/s43705-022-00183-8 (PMC9723582; doi:10.1038/s43705-022-00183-8)
Supplement: Supplementary file 5 — Table S4 [file 43705_2022_183_MOESM5_ESM.pdf]

Table S4

Bacillus Ribosomal Operon(s)	# of Associated RoC-ITS reads	Expected %	Expected #
A	30	0.1	33.6
B	29	0.1	33.6
C	29	0.1	33.6
D	39	0.1	33.6
J	23	0.1	33.6
H	32	0.1	33.6
E	31	0.1	33.6
FGI	123	0.3	100.8
Total	336		
Chi-squared p-value	0.137583828		
