# Supplementary material for: High-resolution phylogenetic and population genetic analysis of microbial communities with RoC-ITS
Source: ISME Commun. 2022 Oct 10;2:99. doi: 10.1038/s43705-022-00183-8 (PMC9723582; doi:10.1038/s43705-022-00183-8)
Supplement: Supplementary file 6 — Table S5 [file 43705_2022_183_MOESM6_ESM.pdf]

Table S5

Duganella Ribosomal Operon(s)	# of Associated RoC-ITS reads	Expected %	Expected #
rrn1	16	0.14	14.1428571
rrn2	21	0.14	14.1428571
rrn3	10	0.14	14.1428571
rrn4	18	0.14	14.1428571
rrn5	6	0.14	14.1428571
rrn6	12	0.14	14.1428571
rrn7	16	0.14	14.1428571
**Total**	99		
**Chi-squared p-value**	0.09		
