# Supplementary figures and images for: High-resolution phylogenetic and population genetic analysis of microbial communities with RoC-ITS
Source: ISME Commun. 2022 Oct 10;2:99. doi: 10.1038/s43705-022-00183-8 (PMC9723582; doi:10.1038/s43705-022-00183-8)

Supplemental Figure 1

Histogram of Nanopore Read Lengths

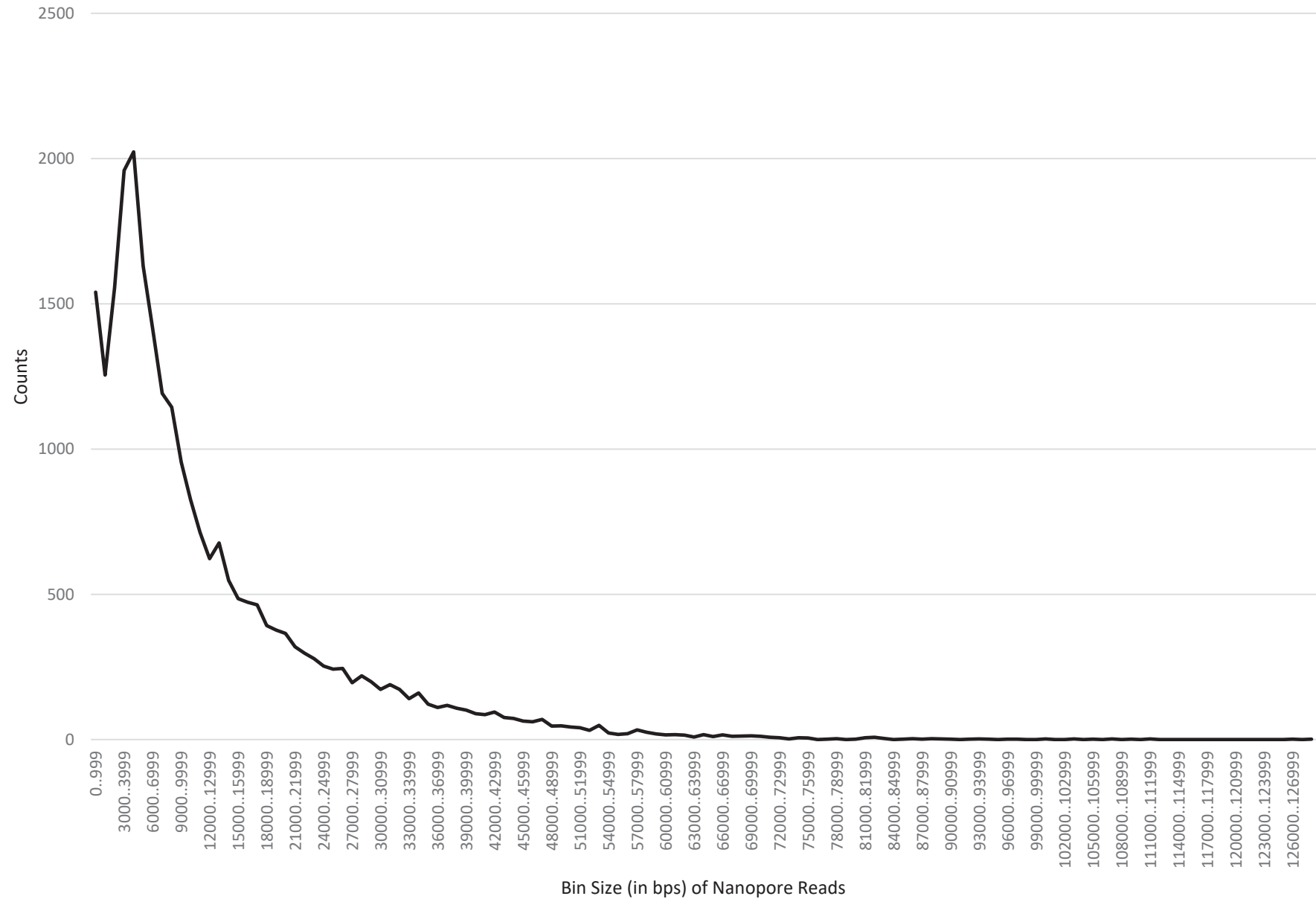

Supplement: Supplementary file 7 — Figure S1 [file 43705_2022_183_MOESM7_ESM.pdf]

Supplemental Figure 2

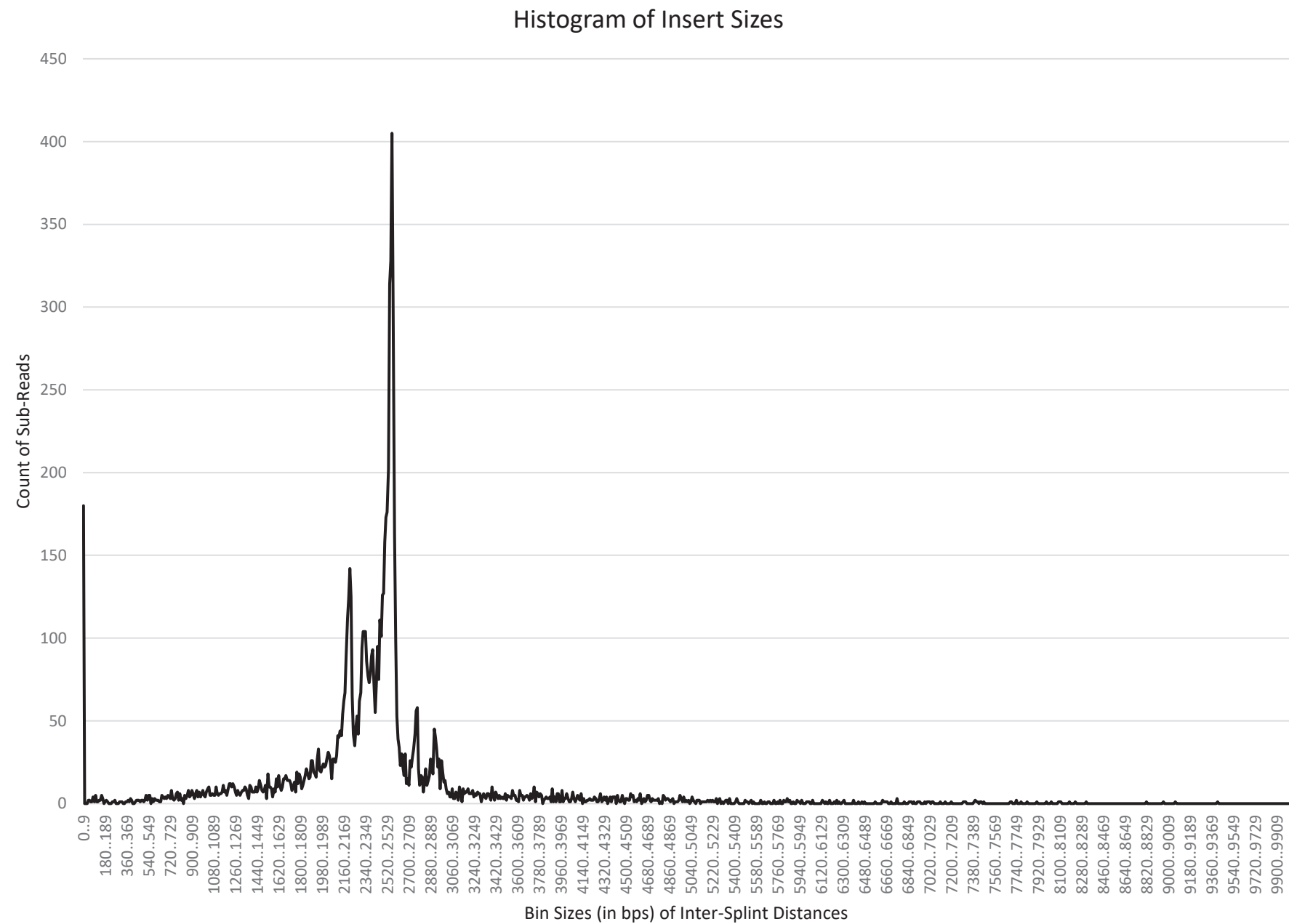

Supplement: Supplementary file 8 — Figure S2 [file 43705_2022_183_MOESM8_ESM.pdf]

# Supplemental Figure 3

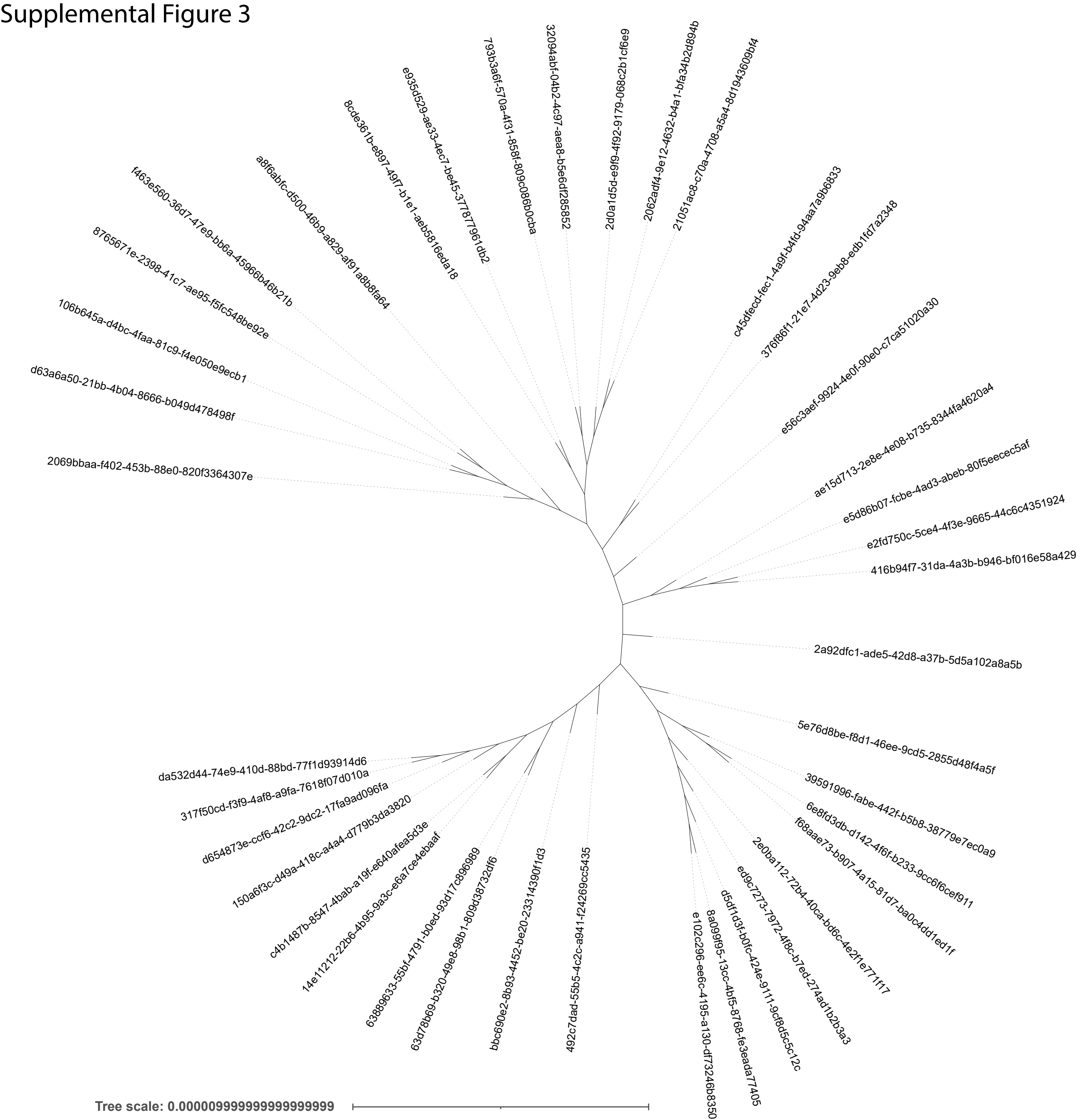

Supplement: Supplementary file 9 — Figure S3 [file 43705_2022_183_MOESM9_ESM.pdf]

## Supplemental Figure 4

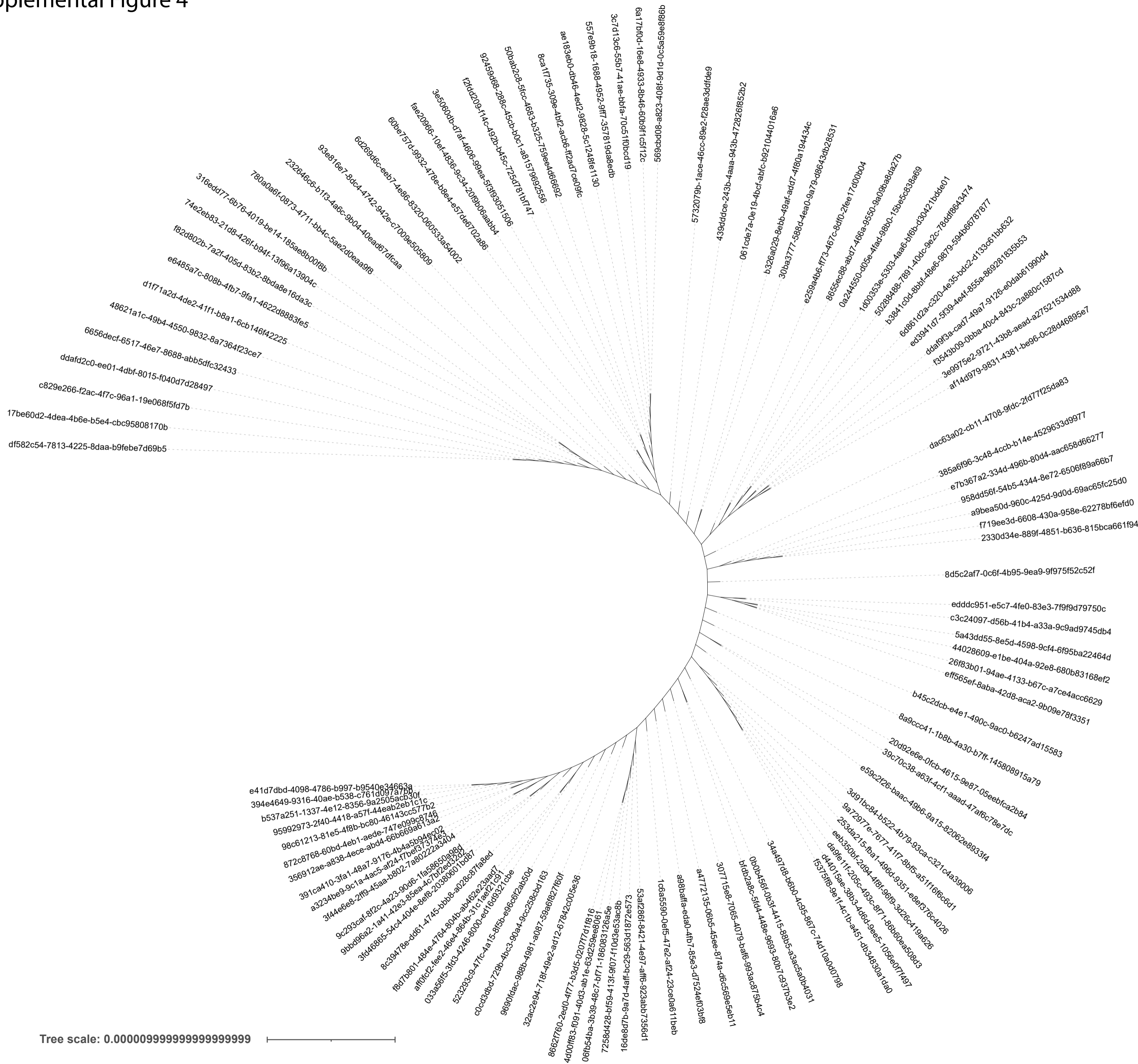

Supplement: Supplementary file 10 — Figure S4 [file 43705_2022_183_MOESM10_ESM.pdf]

Supplemental Figure 5

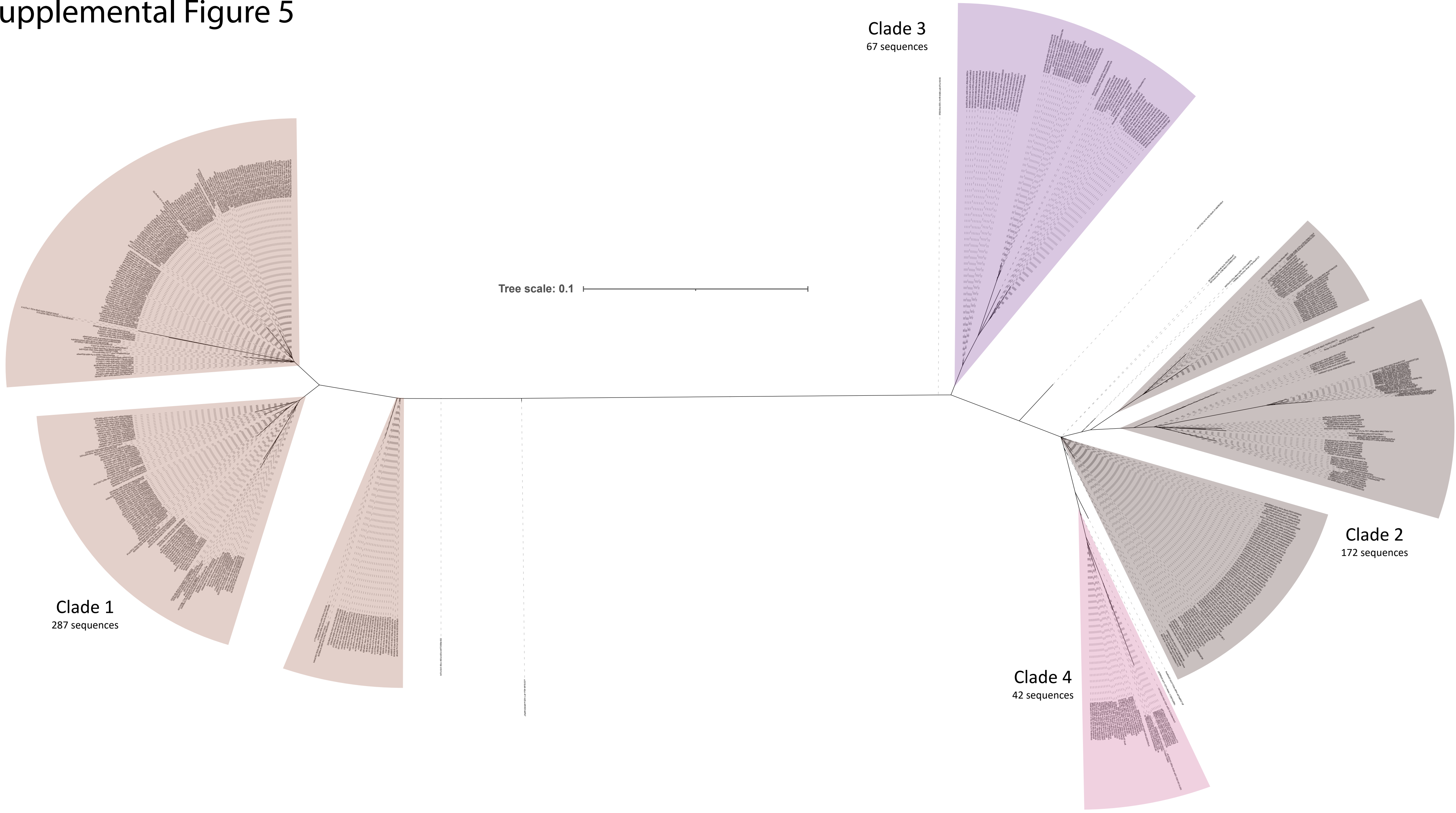

Supplement: Supplementary file 11 — Figure S5 [file 43705_2022_183_MOESM11_ESM.pdf]

Supplemental Figure 6

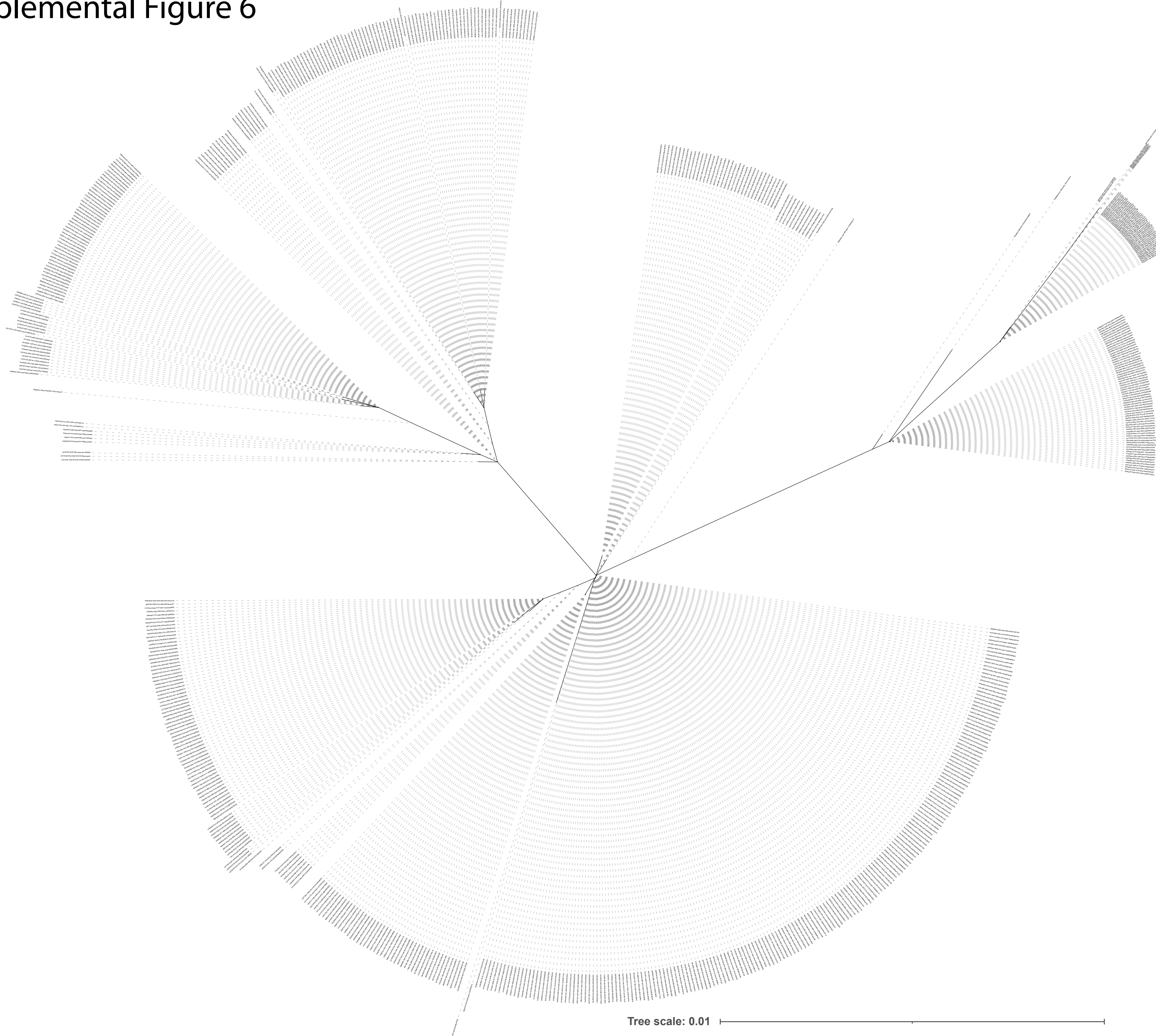

Supplement: Supplementary file 12 — Figure S6 [file 43705_2022_183_MOESM12_ESM.pdf]

# Supplemental Figure 7

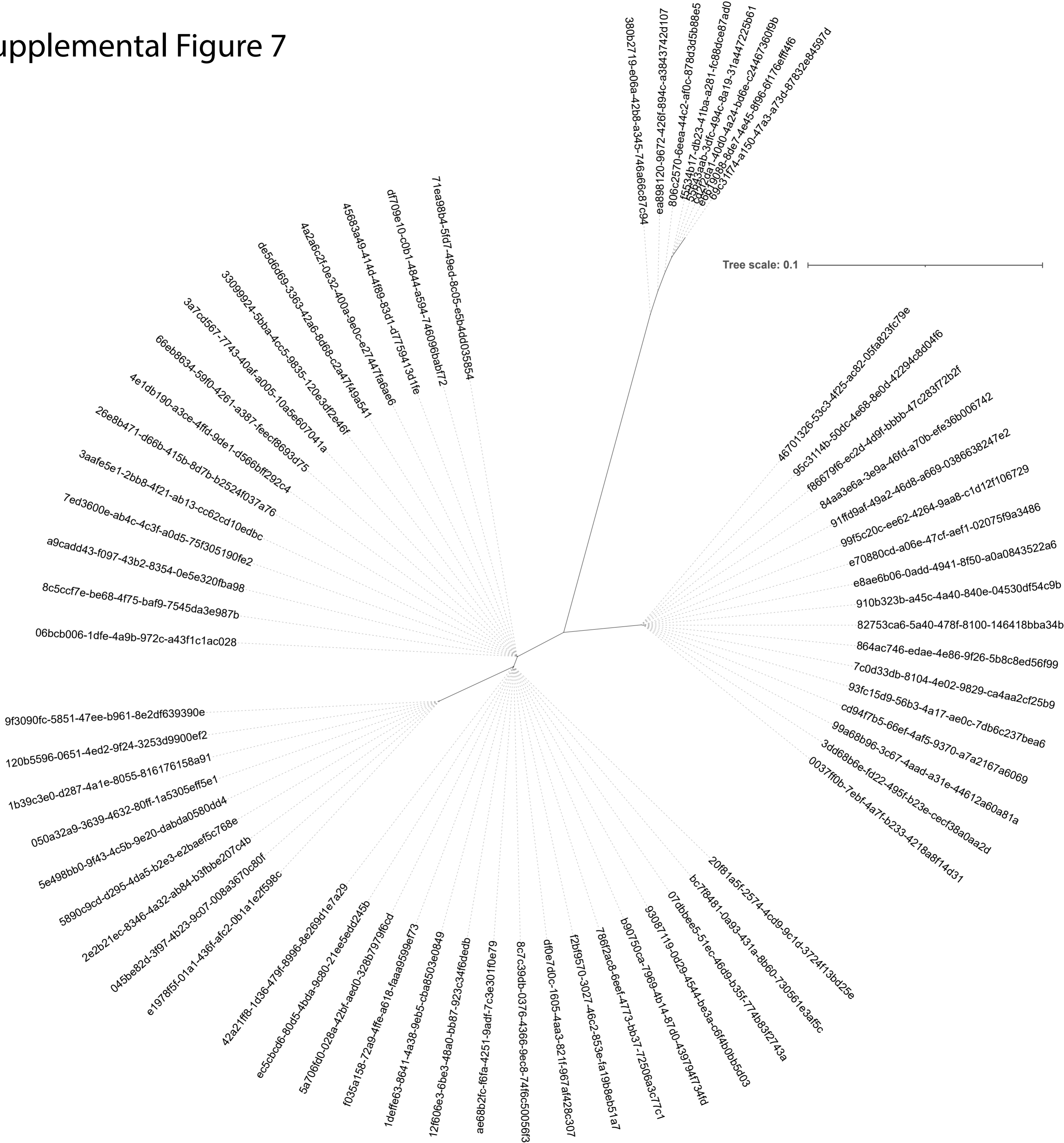

Supplement: Supplementary file 13 — Figure S7 [file 43705_2022_183_MOESM13_ESM.pdf]

# Supplemental Figure 8

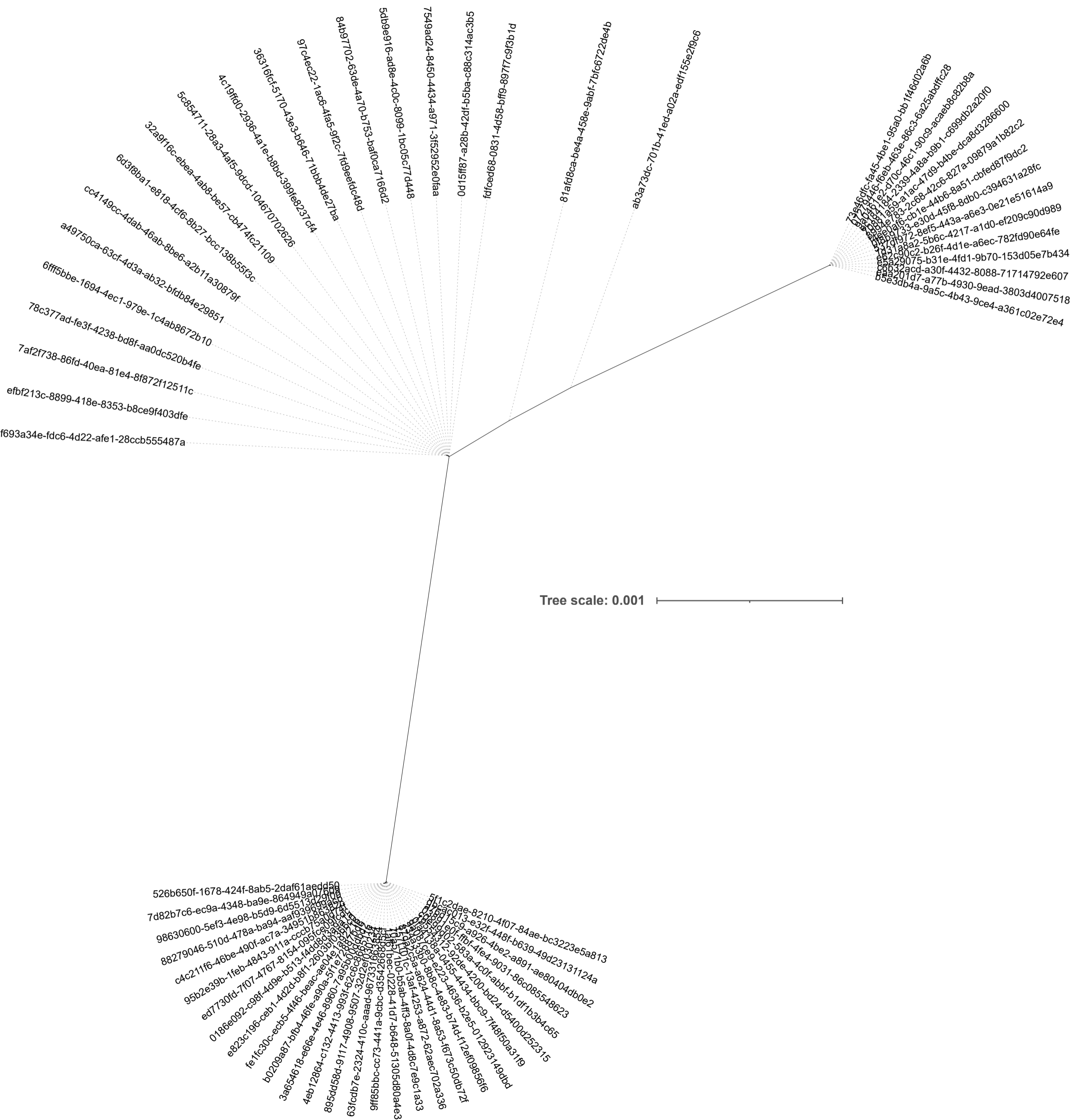

Supplement: Supplementary file 14 — Figure S8 [file 43705_2022_183_MOESM14_ESM.pdf]
